# Cerebrovascular Function in Sporadic and Genetic Cerebral Small Vessel Disease

**DOI:** 10.1002/ana.27136

**Published:** 2024-11-18

**Authors:** Michael S. Stringer, Gordon W. Blair, Anna Kopczak, Danielle Kerkhofs, Michael J. Thrippleton, Francesca M. Chappell, Susana Muñoz Maniega, Rosalind Brown, Kirsten Shuler, Iona Hamilton, Daniela Jaime Garcia, Fergus N. Doubal, Una Clancy, Eleni Sakka, Tetiana Poliakova, Esther Janssen, Marco Duering, Michael Ingrisch, Julie Staals, Walter H. Backes, Robert van Oostenbrugge, Geert Jan Biessels, Martin Dichgans, Joanna M. Wardlaw, The SVDs@target consortium

**Affiliations:** ^1^ Brain Research Imaging Center, Center for Clinical Brain Sciences UK Dementia Institute Center at the University of Edinburgh Edinburgh UK; ^2^ Institute for Stroke and Dementia Research (ISD) University Hospital Munich Germany; ^3^ Department of Neurology, CARIM School for cardiovascular diseases Maastricht University Medical Center Maastricht the Netherlands; ^4^ Medical Image Analysis Center (MIAC AG) and Department of Biomedical Engineering University of Basel Basel Switzerland; ^5^ Department of Radiology University Hospital Munich Germany; ^6^ Department of Radiology & Nuclear Medicine Maastricht University Medical Center, Schools for Mental Health & Neuroscience and Cardiovascular Disease Maastricht the Netherlands; ^7^ Department of Neurology and Neurosurgery, UMC Utrecht Brain Center University Medical Center Utrecht Utrecht Netherlands; ^8^ German Center for Neurodegenerative Diseases (DZNE, Munich) Munich Germany; ^9^ Munich Cluster for Systems Neurology (SyNergy) Munich Germany

## Abstract

**Objective:**

Cerebral small vessel diseases (SVDs) are associated with cerebrovascular dysfunction, such as increased blood–brain barrier leakage (permeability surface area product), vascular pulsatility, and decreased cerebrovascular reactivity (CVR). No studies assessed all 3 functions concurrently. We assessed 3 key vascular functions in sporadic and genetic SVD to determine associations with SVD severity, subtype, and interrelations.

**Methods:**

In this prospective, cross‐sectional, multicenter INVESTIGATE‐SVDs study, we acquired brain magnetic resonance imaging in patients with sporadic SVD/cerebral autosomal dominant arteriopathy with subcortical infarcts and leukoencephalopathy (CADASIL), including structural, quantitative microstructural, permeability surface area product, blood plasma volume fraction, vascular pulsatility, and CVR (in response to CO_2_) scans. We determined vascular function and white matter hyperintensity (WMH) associations, using covariate‐adjusted linear regression; normal‐appearing white matter and WMH differences, interrelationships between vascular functions, using linear mixed models; and major sources of variance using principal component analyses.

**Results:**

We recruited 77 patients (45 sporadic/32 CADASIL) at 3 sites. In adjusted analyses, patients with worse WMH had lower CVR (*B* = −1.78, 95% CI −3.30, −0.27) and blood plasma volume fraction (*B* = −0.594, 95% CI −0.987, −0.202). CVR was worse in WMH than normal‐appearing white matter (eg, CVR: *B* = −0.048, 95% CI −0.079, −0.017). Adjusting for WMH severity, SVD subtype had minimal influence on vascular function (eg, CVR in CADASIL vs sporadic: *B* = 0.0169, 95% CI −0.0247, 0.0584). Different vascular function mechanisms were not generally interrelated (eg, permeability surface area product~CVR: *B* = −0.85, 95% CI −4.72, 3.02). Principal component analyses identified WMH volume/quantitative microstructural metrics explained most variance in CADASIL and arterial pulsatility in sporadic SVD, but similar main variance sources.

**Interpretation:**

Vascular function was worse with higher WMH, and in WMH than normal‐appearing white matter. Sporadic SVD‐CADASIL differences largely reflect disease severity. Limited vascular function interrelations may suggest disease stage‐specific differences. ANN NEUROL 2025;97:483–498

Cerebral small vessel diseases (SVDs) cause one‐quarter of ischemic strokes and up to 50% of dementias, either vascular or mixed.^1^ Sporadic SVD, the commonest type that may be covert, or cause stroke, cognitive impairment, or mobility or mood problems, increases with age.^2^ Genetic SVDs, including cerebral autosomal dominant arteriopathy with subcortical infarcts and leukoencephalopathy (CADASIL), are typically more severe, usually affecting younger adults.

Hypertension is the major modifiable risk factor for sporadic SVD.^3^ However, apart from vascular risk factor management, which has rather limited effect on preventing adverse outcomes,^4^ as yet there are no specific therapies for SVDs, possibly reflecting incomplete understanding of its pathophysiology.^1^

Sporadic and genetic SVDs cause similar types of lesions on brain magnetic resonance imaging (MRI), primarily white matter hyperintensities (WMH), but also lacunes, microbleeds, and increased perivascular space visibility.^5^ SVDs lesions are considered “ischemic” based on pathological studies, but these typically reflect end‐stage damage. Key cerebrovascular mechanisms can be assessed in vivo using MRI, including blood–brain barrier (BBB) leakage using gadolinium‐based contrast agents,^6^ cerebrovascular reactivity (CVR) as the response to CO_2_ during blood oxygen level‐dependent (BOLD) MRI,^7^, ^8^ and venous/arterial pulsatility with phase contrast MRI (PC‐MRI).^9^

In vivo studies in sporadic SVD using these MRI techniques have shown that more severe SVD is associated with subtle BBB leakage,^10^, ^11^ impaired CVR,^12^, ^13^ and higher blood pulsatility index.^9^ However, there have been few studies in genetic SVD,^11^ and no studies investigated these different aspects of cerebrovascular function simultaneously in the same patients.^14^

We established the prospective multisite Imaging NeuroVascular, Endothelial and STructural InteGrity in prepAration to TrEat Small Vessel Diseases (INVESTIGATE‐SVDs) study to determine which of the 3 main vascular function metrics (BBB leakage, CVR, blood pulsatility) were most closely related to SVD severity, and whether the underlying function differed between sporadic SVD and CADASIL. We hypothesized that the 3 cerebrovascular functions would be the most abnormal in patients with the worst WMH burden, and that a similar pattern of more BBB leakage, lower CVR, and higher pulsatility would occur in sporadic SVD and CADASIL.

## Methods

### Regulatory Approvals

INVESTIGATE‐SVDs received ethical approval at Edinburgh (South East Scotland Research Ethics Committee, Reference 16/SS/0123), Maastricht (Medical Ethical Committee of Maastricht University Medical Center, Reference 16–2044), and Munich (Ethics Committee of the LMU Munich, Reference 658–16).^14^ All participants provided written informed consent. INVESTIGATE‐SVDs is registered (ISRCTN 10514229), and followed the STROBE Guidelines.

### Patients

We recruited participants aged ≥18 years with capacity to consent and independent in activities of daily living (modified Rankin score <3) from stroke or specialist genetic SVD clinics who presented with either a lacunar stroke in the past 5 years with a corresponding small subcortical infarct on MRI or computed tomography at presentation, or a formal diagnosis of CADASIL. We excluded participants with other major neurological or psychiatric conditions affecting the brain and interfering with the study design (eg, multiple sclerosis); other causes of stroke (eg, ≥50% luminal stenosis in large arteries supplying the area of ischemia); major‐risk cardioembolic source of embolism; other specific causes of stroke identified (eg, hemorrhage, arteritis, dissection etc.) and other stroke risk factor requiring immediate intervention precluding study participation; and contraindications to MRI, gadolinium‐based contrast agents or CO_2_ challenge (eg, severe respiratory disease).^14^ No healthy control group was acquired, as a healthy control group does not account for medication effects, co‐existing conditions such as hypertension, and the high prevalence of SVD in older age.^1^ Instead, we concentrated on gathering a broad spread of disease burdens. The study protocol, including full inclusion and exclusion criteria, is published elsewhere.^14^

### Clinical Assessment

Before brain MRI, we recorded SVD‐related clinical features (eg, diagnosis date, presenting symptoms, relevant investigations), vascular risk factors (diagnosis of hypertension, hyperlipidemia, diabetes, smoking status), past medical history, and current prescribed medications. We measured resting blood pressure (BP) while seated, pulse, height, and weight.

### Telemetric BP

All participants recorded their BP at home for 7 days before MRI using a validated, CE‐marked telemetric BP device (Tel‐O‐Graph; IEM GmbH, Stolberg, Germany),^15^ taking 2 consecutive readings while seated 3 times per day (on waking, at midday, and before bed).^14^ Readings were transferred telemetrically to a central database in Munich.

We calculated BP variability (BPV)^16^, ^17^ from the telemetric BP data as the coefficient of variation (standard deviation/mean) using second readings on waking, around lunch time, and before bed, using an in‐house MATLAB script. Full details have been previously described.^14^, ^18^

### MRI Acquisition

Participants underwent the same structural and vascular function 3 Tesla brain MRI protocol at all 3 sites on Siemens Prisma scanners (Siemens Healthcare, Erlangen, Germany; apart from the first 3 scans in Munich, which were acquired on a Siemens Skyra). Full details are published,^14^, ^18^ including the quality assurance program (Supplementary Methods S1 and Table 1), and can be downloaded at https://harness-neuroimaging.org. All imaging protocols^7^, ^9^, ^19^, ^20^ followed consensus recommendations.^6^ The protocol included:

- Structural imaging 3D T1‐weighted, T2‐weighted (T2‐w), fluid attenuated inversion recovery, and susceptibility‐weighted imaging to assess disease burden and measure brain volumes;
- Multi‐shell diffusion imaging (dMRI) to quantify white matter microstructure;
- Quantitative *T*_*1*_ relaxation time to assess tissue water content, and to use in the BBB permeability calculations;
- Dynamic contrast‐enhanced MRI (DCE‐MRI) with 0.1 mmol/kg bodyweight intravenous gadobutrol (1 M Gadovist; Bayer AG, Leverkusen, Germany) to assess BBB leakage (permeability surface area product [PS]) and blood plasma volume fraction (*v*_*P*_);
- Phase contrast (PC) MRI to assess blood flow and pulsatility in the internal carotid and vertebral arteries, internal jugular veins, straight, sagittal and transverse venous sinuses, and cerebrospinal fluid at the foramen magnum;
- Dynamic BOLD sequence during alternating inspiration of 2 minutes medical air (21:79 O_2_:N_2_) and 3 minutes 6% CO_2_ (balance 21:73 O_2_:N_2_, 2 cycles) delivered from gas cylinders to measure CVR using a proven reproducible paradigm suitable for patients with SVD that gives a robust response of cerebral microvessels while allowing natural, unforced respiration.^7^, ^18^

**Table 1:** Demographics, Blood Pressure, Small Vessel Diseases Lesion Visual Ratings, and Structural Brain Volumes.

	All patients	Sporadic SVD	CADASIL	Sporadic SVD versus CADASIL
**Demographics**				
Total, n (%)	77 (100)	45 (100)	32 (100)	
M/F, n (%)	42/35 (54.5/45.5)	26/19 (57.7/42.2)	16/16 (50.0/50.0)	χ^2^ = 0.456, *p* = 0.50
Age (yr)	59.5 ± 12.3 (23.6–87.0)	64.2 ± 11.0 (43.0–87.0)	52.9 ± 11.1 (23.6–70.0)	*t* = 4.44, 95% CI = 6.24, 16.4
Diabetes	10 (13.0)	9 (20.0)	1 (3.1)	*p* = 0.039
Hypertension	46 (59.7)	35 (77.7)	11 (34.4)	χ^2^ = 14.6, *p* < 0.001
Hyperlipidemia	46 (59.7)	33 (73.3)	13 (40.6)	χ^2^ = 8.32, *p* = 0.004
Current & ex‐smoker	41 (53.3)	23 (51.1)	18 (56.3)	χ^2^ = 0.198, *p* = 0.66
Does use alcohol	55 (71.4)	31 (68.8)	24 (75.0)	χ^2^ = 0.342, *p* = 0.56
Alcohol units /week	1 (0–5)	2 (0–7)	1 (0.5–2.5)	*t* = 1.01, 95% CI = −9.67, 2.97
**Blood pressure**				
Pre‐CVR systolic (mmHg)	143.6 ± 24.3 (90.0–200.0)	156.7 ± 23.2 (90.0–200.0)	127.7 ± 13.9 (110.0–160.0)	*t* = 6.53, 95% CI = 20.1, 37.9
Pre‐CVR diastolic (mmHg)	82.2 ± 12.3 (50.0–110.0)	86.7 ± 12.7 (50.0–110.0)	76.7 ± 9.5 (60.0–90.0)	*t* = 3.81, 95% CI = 4.78, 15.3
Mean 24‐h systolic (mmHg)	125.0 ± 12.3 (100.7–157.1)	130.3 ± 11.6 (105.7–157.1)	117.5 ± 9.1 (100.7–139.9)	*t* = 5.40, 95% CI = 8.04, 17.4
Mean 24‐h diastolic (mmHg)	80.4 ± 9.5 (60.6–105.4)	83.0 ± 9.13 (62.4–105.4)	76.7 ± 8.9 (60.6–90.2)	*t* = 3.08, 95% CI = 2.24, 10.5
Systolic BPV (unit less)	0.0750 ± 0.0229 (0.0321–0.1493)	0.0821 ± 0.0241 (0.0340–0.1493)	0.0651 ± 0.0169 (0.0321–0.1063)	*t* = 3.66, 95% CI = −0.008, 0.0264
Diastolic BPV (unit less)	0.0801 ± 0.0235 (0.0362–0.1444)	0.0804 ± 0.0214 (0.0488–0.1444)	0.0796 ± 0.0266 (0.0362–0.1400)	*t* = 0.14, 95% CI = −0.011, 0.012
**Visual SVD ratings**				
Total Fazekas	4 (3–6)	3 (2–4)	6 (5–6)	*W* = 1856, *p* < 0.0001
Total PVS	5 (3–6)	4 (3–5)	6 (4–8)	*W* = 1,641, *p* = 0.0001
No. of lacunes	3 (0–7)	1 (0–4)	5.5 (1–9)	*W* = 1,524, *p* = 0.0051
No. of microbleeds	0 (0–4)	0 (0–1)	1.5 (0–8)	*W* = 1,474, *p* = 0.0134
Deep atrophy score	3 (3–4)	4 (3–5)	3 (2–3)	*W* = 1,007, *p* = 0.012
Superficial atrophy score	3 (3–4)	4 (3–4)	3 (2–4)	*W* = 1,103, *p* = 0.13
Intracranial volume (ml)	1413.03 ± 133.37 (1142.07–1878.32)	1425.49 ± 151.52 (1180.12–1878.32)	1395.51 ± 102.39 (1142.07–1603.28)	*t* = 1.04, 95% CI = −27.7, 87.6
Brain volume (ml)	1104.92 ± 101.29 (881.47–1360.20)	1085.60 ± 108.63 (881.47–1307.42)	1132.08 ± 84.26 (963.88–1360.2)	*t* = −2.11, 95% CI = −90.3, −2.64
CSF volume (ml)	302.38 ± 74.54 (172.04–609.08)	334.35 ± 71.23 (219.21–609.08)	257.42 ± 53.37 (172.04–362.92)	*t* = 5.42, 95% CI = 48.6, 105
GM volume (ml)	530.64 ± 51.39 (411.65–679.04)	536.00 ± 53.04 (421.61–679.04)	523.09 ± 48.80 (411.65–629.02)	*t* = 1.10, 95% CI = −10.4, 36.3
NAWM volume (ml)	532.76 ± 57.96 (421.07–701.99)	537.53 ± 55.79 (432.54–655.42)	526.05 ± 61.14 (421.07–701.99)	*t* = 0.84, 95% CI = −15.8, 38.7
WMH volume (ml)	14.63 (5.71–58.24)	7.94 (4.24–11.97)	69.88 (40.13–113.30)	*W* = 1884, *p* < 0.0001

All values reported as number (percentage) for categorical, mean ± standard deviation (range) for normally distributed numeric variables, and median (interquartile range) otherwise. χ^2^ for chi‐squared, *p* for *p* value, *t* for *t* value, 95% CI for 95% confidence interval and *W* for Wilcoxon's rank sum. Fisher's exact test was used for diabetes. All statistical tests are unadjusted for covariates.

Abbreviations: BPV = blood pressure variability; CADASIL = cerebral autosomal dominant arteriopathy with subcortical infarcts and leukoencephalopathy; CSF = cerebrospinal fluid; CVR = cerebrovascular reactivity; GM = gray matter; NAWM = normal‐appearing white matter; SVD = small vessel disease; WMH = white matter hyperintensity.

### MRI Quality Assurance

We performed regular quality assurance using phantoms and volunteers throughout the study to monitor scanner stability and acquisitions, and ensure data consistency (see Supplementary Methods/Quality assurance).

### MRI Processing and Analysis

All imaging data were anonymized and transferred securely to Edinburgh using established protocols (https://www.ed.ac.uk/clinical-sciences/edinburgh-imaging/research/services-and-collaboration/smartis). All analyses used validated methods,^14^ were blinded to all other measures, and visually checked for accuracy, briefly summarized here (full details of image processing including region of interest determination, dMRI, *T*_*1*_ relaxation time, and vascular function measures are in Supplementary Materials and the published protocol^14^).

#### SVD Features Visual Assessment

We rated structural images for SVD features using the STRIVE‐1 criteria^5^ (E.J., J.M.W.). We scored WMHs using the Fazekas scale,^21^ summing periventricular and deep WMH scores to give a score from 0 to 6; perivascular spaces (PVS) using a validated, semiquantitative ordinal scale (range 0–4) summing basal ganglia and centrum semiovale scores^5^; presence/absence and total number of microbleeds; and determined brain atrophy score (range 1–6) with reference to a normal aging template.^22^

#### Whole and Subregional Brain and WMH Volumes Segmentation

We co‐registered structural images to the T2‐w images using FLIRT.^23^ We determined intracranial volume by extracting^24^ the brain from the magnitude susceptibility‐weighted imaging. We (M.S.) manually delineated and excluded stroke lesions according to STRIVE‐1 guidelines^5^ with neuroradiological supervision (J.M.W.).

We assessed vascular function in normal‐appearing white matter (NAWM), subcortical gray matter (SGM), and WMH regions of interest. We segmented SGM using FIRST,^25^ combining the caudate, putamen, pallidum, and thalamus. We applied a validated semiautomatic technique to calculate WMH volumes based on intensity thresholding and a multispectral approach, and excluded stroke lesions.^9^ We segmented whole‐brain NAWM and cerebrospinal fluid (including ventricles), using FAST.^26^ We excluded WMH, SGM, brainstem, and stroke lesion masks from the NAWM mask, and WMH and stroke lesions from the SGM mask.

We eroded the SGM and NAWM masks inwards by 2 mm circumferentially to reduce partial volume effects while maximizing tissue retention. We did not erode the WMH mask to avoid excluding small punctate hyperintensities. As vessels running on the inner ventricular surface artefactually increase the BOLD signal, we dilated the ventricles to exclude adjacent periventricular tissue (whether NAWM or WMH) by 5 voxels (5 mm) left–right, and 4 voxels (4 mm) infero‐superior and antero‐posterior. For each participant, we registered and overlaid the resulting masks on the voxelwise CVR map to exclude “blooming artefacts” from large veins and venous sinuses. All masks were checked and manually edited as needed to avoid misclassification (see Supplementary Methods image processing).

#### Quantitative Tissue and Vascular Function Metrics

Using validated established techniques, we processed the dMRI, quantitative *T*_1_,^19^, ^27^ DCE‐MRI, PC‐MRI, and CVR^6^, ^7^, ^9^ data for each region of interest (details in supplement). We did not perform voxelwise analyses, as the contrast‐to‐noise ratio for the BOLD and DCE‐MRI^6^, ^7^ signals are generally low.

### Statistical Analysis

We reported all data for all available participants. We calculated summary statistics as the mean ± standard deviation and (range)/median (interquartile range) for normally/non‐normally distributed continuous data respectively, and proportions for count data. We used histograms/bar charts for univariate plots and scatterplots for bivariate relationships. We used log_10_ of WMH volume standardized to intracranial volume to account for head size and meet linear regression assumptions. For unadjusted (univariate) comparisons, we reported χ^2^‐tests as χ^2^, 95% confidence intervals (95% CI), *p* values for binary/unpaired categorical data, and used Fisher's exact test (*p*‐value) where there were insufficient values (<5); *t* tests as *t* value, 95% CI, *p* value for continuous variables; and Wilcoxon's rank sum, *p*‐value for non‐parametric data.

We report descriptive results for BP measures and diagnosis of hypertension. Based on previous studies,^16^, ^17^ we used systolic BPV in analyses.

We used multivariable linear regression to assess the association between WMH volume and vascular function metrics measured in NAWM and WMH, using separate models adjusted for key vascular risk factors (age, smoking status [0 = never, 1 = current/ever smoker], SVD subtype, and systolic BPV). We chose these risk factors as they are known important WMH predictors. We limited the number of risk factors to avoid overfitting and poor generalizability.^28^ We report unstandardized coefficients (*B*), 95% CI, and *p* value.

We used linear mixed models to examine how vascular function metrics measured in NAWM and WMH interrelated and differed between the two tissues adjusted for the aforementioned co‐variates (age, smoking status, SVD subtype, and systolic BPV), the remaining vascular function metrics, with an interaction term for tissue type (NAWM/WMH) and WMH volume, with participant ID as a random effect. We tested CVR, *v*_*P*_ and BBB PS as the outcomes. The coefficient for outcome~WMH volume is given for NAWM; to obtain the coefficient for WMH, the interaction term effect is added. Excluding WMH volume would have biased the coefficient estimates. For all models, we considered the direction of effect of the point estimate, breadth of the confidence interval, and existing clinical knowledge rather than solely *p* values when assessing and interpreting relationships between variables.^29^, ^30^

Finally, we conducted a principal components analysis (PCA), an unsupervised data‐reduction technique that seeks to identify the main sources of variance in the data by grouping related variables into “latent factors” that each explain part of the variance in the data.^31^ Each “latent factor” was then ranked according to the proportion of variance it explained in the whole dataset. We report only factors that explained more variance than random noise, decided using scree and parallel line plots. We examined the whole dataset first, then examined the CADASIL and sporadic SVD participants separately in sensitivity analyses.

For all analyses, we checked underlying statistical assumptions and removed predictors causing collinearity, as assessed using variance inflation factors, where necessary. We rescaled mean diffusivity (MD; ×1,000), PS (×10,000), and *v*_*P*_ (×100) for range consistency with other variables to avoid introducing collinearity.

We used SAS 9.4 (www.sas.com) for regression analyses and PCA, and R 3.6.2 (https://cran.r-project.org) for graphs.

## Results

### Patient Characteristics

We recruited 77 patients; 45 with sporadic SVD (25 at Edinburgh, 20 at Maastricht) and 32 with CADASIL (at Munich). All patients provided complete, analyzable structural imaging, but 7 CVR, 4 dMRI, 1 PC‐MRI, 2 quantitative *T*_1_, and 9 DCE‐MRI scans were not analyzable (reasons in Supplementary Figure S2).

The 77 patients had a mean age of 59.5 ± 12.3 years (23.6–87.0 years), 35 were women, 60% had hypertension, 60% hyperlipidemia, 13% diabetes, and 53% were current/ex‐smokers (Table 1). Patients with sporadic SVD were older (*t* = 4.4, 95% CI 6.2, 16.4, *p* < 0.001) and more often had diabetes (Fisher's, 95% CI 3.7, 30.0, *p* = 0.039), hypertension (χ^2^ = 14.6, 95% CI 22.9, 63.9, *p* < 0.001), and hyperlipidemia (χ^2^ = 8.3, 95% CI 11.3, 54.1, *p* = 0.004) than those with CADASIL.

The mean telemetric BP was 125.0 ± 12.3 mmHg (100.7–157.1 mmHg) systolic and 80.4 ± 9.5 mmHg (60.6–105.4 mmHg) diastolic. Systolic BP (*t* = 5.4, 95% CI 8.0–17.4, *p* < 0.0001), diastolic BP (*t* = 3.1, 95% CI 2.2, 10.5, *p* = 0.003), systolic BPV (*t* = −0.0171, 95% CI 0.0078, 0.0264, *p* = 0.0005), and MRI visit BP measurements (Table 1) were higher in patients with sporadic SVD than CADASIL.

**Figure 1:**
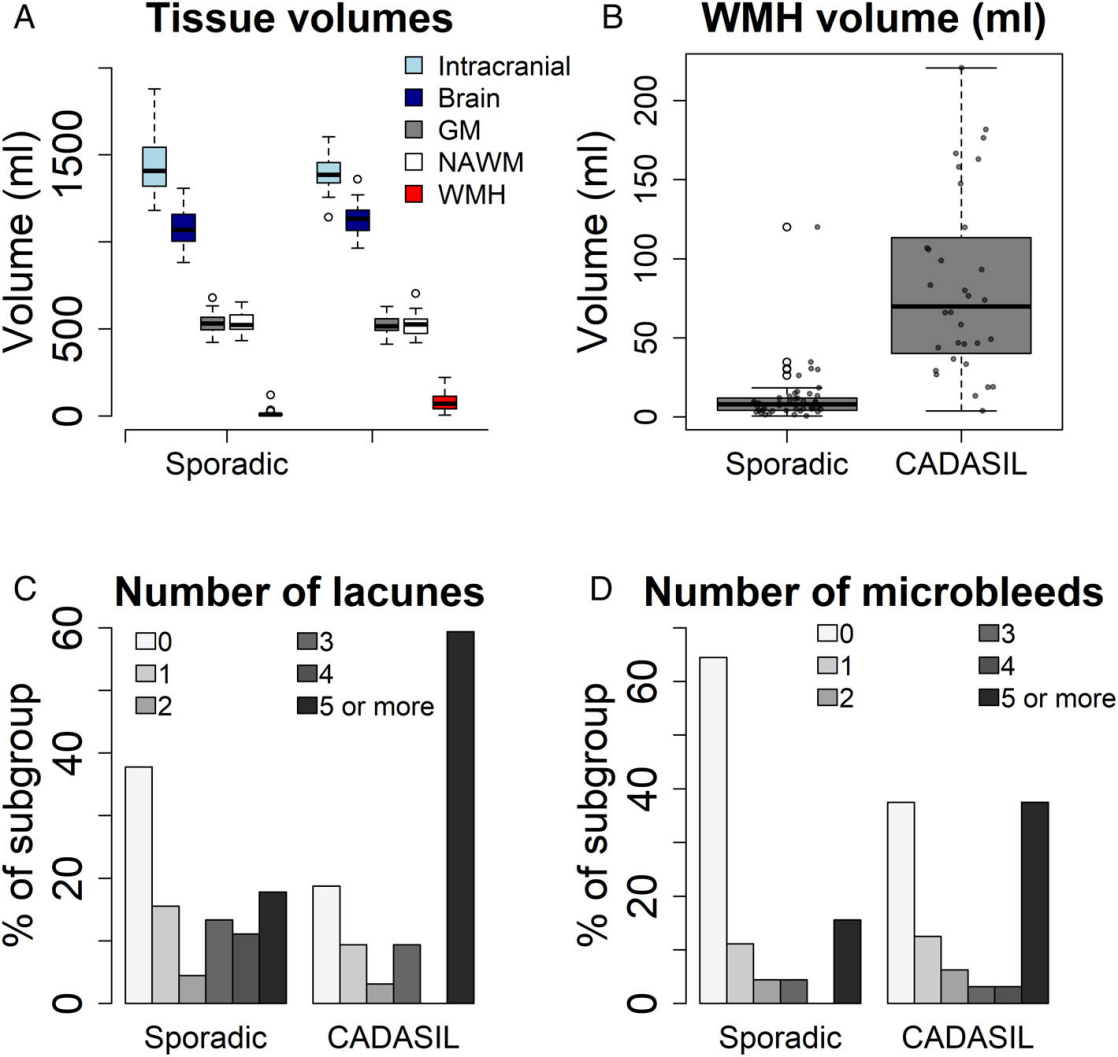
Panel showing key imaging characteristics by small vessel disease subtype. (A) Tissue volumes (ml), (B) white matter hyperintensity (WMH) volume (ml), (C) number of lacunes, and (D) number of microbleeds. WMH, white matter hyperintensity volume. CADASIL = cerebral autosomal dominant arteriopathy with subcortical infarcts and leukoencephalopathy; GM = gray matter; NAWM = normal‐appearing white matter.

The median total Fazekas score was 4 (3–6), 48 patients (62%) had moderate–severe WMH (Fazekas ≥4), the median total PVS score was 5 (3–6), and number of lacunes 3 (0–7) and microbleeds was 0 (0–4); 54 (70%) patients had lacunes and 36 (47%) had microbleeds. All SVD features were worse in patients with CADASIL than sporadic SVD (Table 1, Fig 1 and S3); for example, WMH volume CADASIL 69.9 ml (40.1–113.3 ml), sporadic SVD 7.94 ml (4.2–12.0ml); Wilcoxon 1884, *p* < 0.0001.

Vascular function and tissue structural imaging measures are shown in Table 2. In unadjusted comparisons, some measures were worse in patients with CADASIL than sporadic SVD; for example, MD was higher, fractional anisotropy (FA) and *v*_*P*_ lower in NAWM and WMH, and venous pulsatility lower in CADASIL versus sporadic SVD patients.

**Table 2:** Vascular Function, Quantitative *T*_*1*_ and Diffusion Imaging Metrics for All Patients and by Disease Subtype.

	All patients	Sporadic SVD	CADASIL	CADASIL versus sporadic SVD
**Permeability surface area (10** ^**−4**^ **min** ^**−1**^)				
Subcortical gray matter	0.90 ± 1.19 (−3.52–4.56)	0.97 ± 1.30 (−3.52–4.56)	0.80 ± 1.01 (−1.88–2.85)	*t* = 0.62, 95% CI = −0.38, 0.73
Normal‐appearing white matter	0.25 ± 0.91 (−2.39–2.01)	0.23 ± 0.97 (−2.39–2.01)	0.29 ± 0.83 (−1.21–1.81)	*t* = −0.28, 95% CI = −0.50, 0.37
White matter hyperintensity	0.79 ± 1.05 (−2.39–3.14)	0.71 ± 1.17 (−2.39–3.14)	0.92 ± 0.84 (−1.57–2.63)	*t* = −8.71, 95% CI = −0.70, 0.27
**Plasma volume** (*v*_P_, 10^−2^)				
Subcortical gray matter	1.27 ± 0.28 (0.62–1.79)	1.34 ± 0.25 (0.81–1.77)	1.17 ± 0.29 (0.62–1.79)	*t* = 2.59, 95% CI = 0.04, 0.31
Normal‐appearing white matter	0.55 ± 0.18 (0.12–0. 96)	0.63 ± 0.16 (0.35–0.96)	0. 44 ± 0. 14 (0. 12–0. 73)	*t* = 5.08, 95% CI = 0.12, 0.27
White matter hyperintensity	0.67 ± 0.28 (0.21–1.76)	0.76 ± 0.29 (0.22–1.76)	0.54 ± 0.21 (0. 21–1.16)	*t* = 3.64, 95% CI = 0.10, 0.34
**Phase contrast MRI**				
Arterial pulsatility index	1.25 ± 0.35 (0.56–2.90)	1.23 ± 0.41 (0.56–2.90)	1.27 ± 0.25 (0.89–2.04)	*t* = −0.54, 95% CI = ‐0.19, 0.11
Superior sagittal sinus pulsatility index	0.47 ± 0.18 (0.10–0.95)	0.51 ± 0.18 (0.21–0.95)	0.41 ± 0.15 (0.10–0.76)	*t* = 2.54, 95% CI = 0.02, 0.17
Cerebrospinal fluid stroke volume at foramen magnum (ml)	0.57 ± 0.24 (0.10–1.64)	0.59 ± 0.28 (0.10–1.64)	0.55 ± 0.20 (0.11–0.85)	*t* = 0.58, 95% CI = −0.08, 0.14
**Cerebrovascular reactivity (%/mmHg**)				
Subcortical gray matter	0.127 ± 0.072 (−0.182–0.237)	0.132 ± 0.074 (−0.182–0.237)	0.121 ± 0.071 (−0.101–0.220)	*t* = 0.65, 95% CI = −0.024,0.046
Normal‐appearing white matter	0.035 ± 0.036 (−0.128–0.093)	0.035 ± 0.036 (−0.128–0.086)	0.035 ± 0.036 (−0.058–0.093)	*t* = −0.06, 95% CI = −0.018, 0.017
White matter hyperintensity	0.022 ± 0.071 (−0.284–0.167)	0.022 ± 0.082 (−0.284–0.167)	0.021 ± 0.056 (−0.138–0.114)	*t* = 0.05, 95% CI = −0.033,0.034
***T***_***1***_ (**s**)				
Subcortical gray matter	1.26 ± 0.07 (1.15–1.53)	1.25 ± 0.04 (1.19–1.36)	1.28 ± 0.08 (1.15–1.53)	*t* = −2.03, 95% CI = −0.07, −0.00
Normal‐appearing white matter	0.94 ± 0.04 (0.87–1.05)	0.94 ± 0.04 (0.88–1.04)	0.95 ± 0.04 (0.87–1.05)	*t* = −1.18, 95% CI = −0.03, 0.01
White matter hyperintensity	1.35 ± 0.11 (1.14–1.75)	1.34 ± 0.12 (1.14–1.75)	1.38 ± 0.10 (1.22–1.59)	*t* = −1.50, 95% CI = −0.09, 0.01
**Mean diffusivity (10** ^**−3**^ **mm** ^**2**^ **/s**)				
Subcortical gray matter	0.70 ± 0.08 (0.54–1.01)	0.66 ± 0.035 (0.62–0.80)	0.74 ± 0.09 (0.54–1.01)	*t* = −4.68, 95% CI = −0.11, −0.04
Normal‐appearing white matter	0.65 ± 0.03 (0.58–0.70)	0.63 ± 0.03 (0.58–0.70)	0.66 ± 0.03 (0.60–0.70)	*t* = −4.72, 95% CI = −0.05, −0.02
White matter hyperintensity	0.95 ± 0.11 (0.69–1.20)	0.90 ± 0.09 (0.69–1.16)	1.02 ± 0.08 (0.87–1.20)	*t* = −6.16, 95% CI = −0.17, −0.09
**Fractional anisotropy**				
Subcortical gray matter	0.24 ± 0.03 (0.16–0.32)	0.25 ± 0.02 (0.20–0.32)	0.22 ± 0.030 (0.16–0.28)	*t* = 4.54, 95% CI = 0.02, 0.04
Normal‐appearing white matter	0.48 ± 0.03 (0.37–0.54)	0.50 ± 0.02 (0.45–0.54)	0.46 ± 0.03 (0.37–0.51)	*t* = 6.27, 95% CI = 0.03, 0.06
White matter hyperintensity	0.31 ± 0.06 (0.20–0.42)	0.34 ± 0.04 (0.24–0.42)	0.27 ± 0.04 (0.20–0.41)	*t* = 7.27, 95% CI = 0.05, 0.09

All values reported as mean ± standard deviation (range). Differences between CADASIL and sporadic SVD presented as *t*‐tests: *t*‐value and 95% confidence intervals (95% CI), unadjusted for covariates.

Abbreviations: CADASIL = cerebral autosomal dominant arteriopathy with subcortical infarcts and leukoencephalopathy; MRI = magnetic resonance imaging; SVD = small vessel disease.

### WMH Volume and Vascular Functions

**Figure 2:**
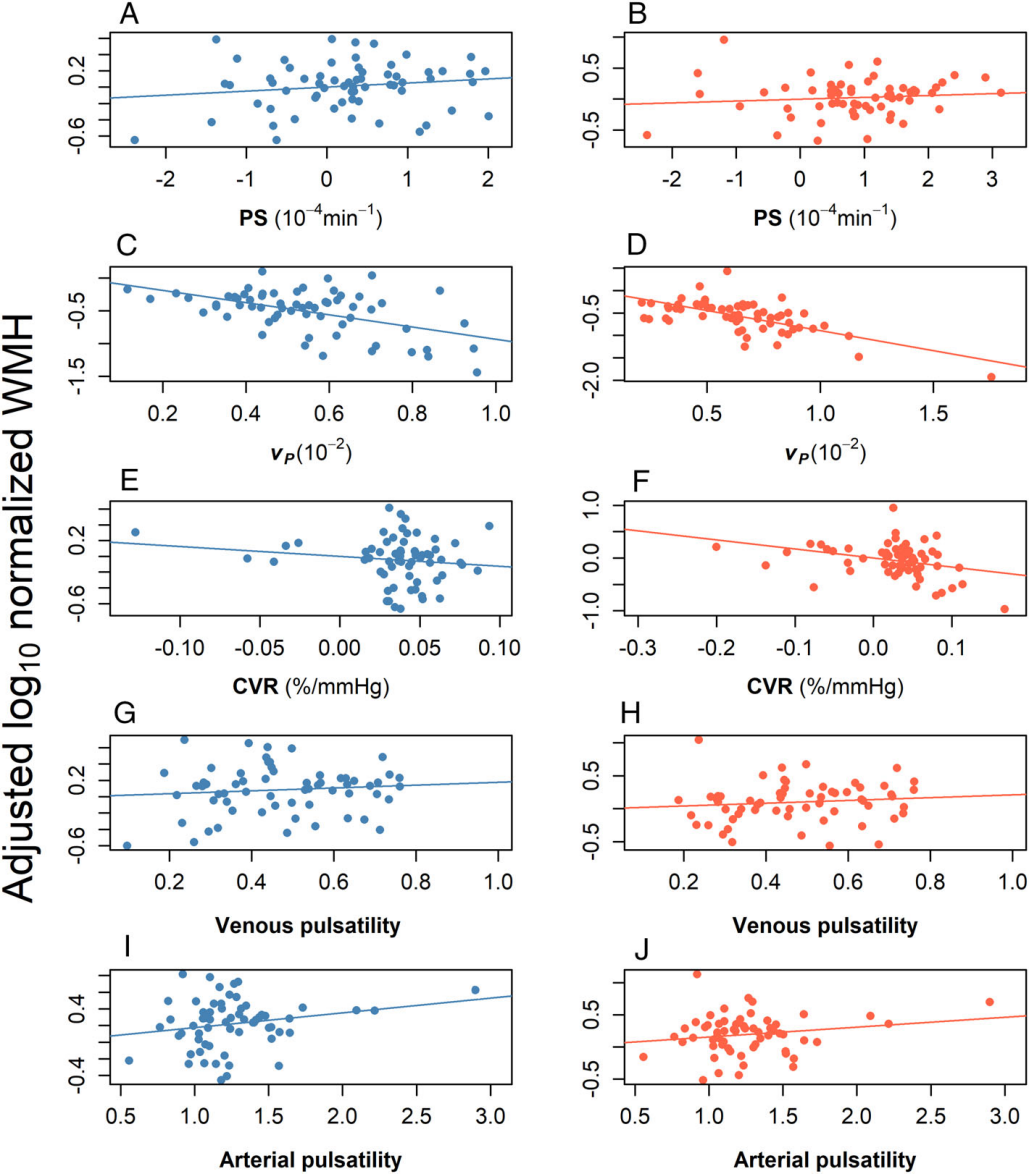
Graphs showing regression lines (see Table 3 for details of coefficients) showing log_10_ normalized white matter hyperintensity (WMH) volume, adjusted for age, smoking status, systolic blood pressure, and the remaining imaging variables against each tissue/vascular function in normal‐appearing white matter (blue, left) and WMH (red, right) for (A, B) permeability surface area product (PS); (C, D) blood plasma volume (*v*_*P*_); (E, F) cerebrovascular reactivity (CVR); (G, H) venous pulsatility; and (I, J) arterial pulsatility. Blood plasma volume fraction (*v*_*P*_) was substituted for PS, and arterial pulsatility for venous pulsatility in separate models, as the variables were derived from the same data source or were collinear, and to avoid overspecifying the model.

Patients with higher WMH volumes had lower *v*_*P*_ (*B* = −0.594, 95% CI −0.987, −0.202, *p* = 0.0037), lower CVR (*B* = 1.78, 95% CI −3.30, −0.27, *p* = 0.02), and a tendency to higher arterial (*B* = 0.119, 95% CI −0.127, 0.365, *p* = 0.34), venous pulsatility (*B* = 0.116, 95% CI −0.567, 0.799, *p* = 0.73), and higher PS (*B* = 0.010, 95% CI −0.075, 0.095, *p* = 0.82) in WMH, with a broadly similar pattern in NAWM (Table 3, Fig 2).

**Table 3:** Linear Regressions with Outcome White Matter Hyperintensity Volume Log(base 10) Normalized to Intracranial Volume Against Tissue‐Specific Predictors

Outcome	Log_10_ (WMH volume/intracranial volume)		
	**Tissue‐specific predictors in NAWM**		
**Predictor**	*B*	95% CI	*p* value
Intercept	−2.73	−3.47 to −1.99	<0.0001
Age	0.0134	0.0032–0.0236	0.011
Smoker	0.102	−0.086–0.289	0.28
PS (×10,000)	0.020	−0.087–0.126	0.71
CVR	−1.72	−4.65–1.20	0.24
CADASIL vs sporadic	0.99	0.76–1.22	<0.0001
Systolic BP variability	−6.44	−11.2 to −1.72	0.0084
Venous pulsatility index	0.224	−0.487–0.936	0.53
Plasma volume (*v*_*P*_, ×100)	−0.589	−1.19–0.01	0.054
Arterial pulsatility index	0.116	−0.154–0.386	0.39
	**Tissue‐specific predictors in WMH**		
Intercept	−2.6	−3.3 to −1.9	<0.0001
Age	0.0104	−0.000 –0.0203	0.041
Smoker	0.096	−0.080–0.272	0.28
PS (×10,000)	0.010	−0.07 –0.095	0.82
CVR	−1.78	−3.30 to −0.27	0.02
CADASIL vs sporadic	0.98	0.78–1.19	<0.0001
Systolic BP variability	−5.00	−9.37 to −0.63	0.026
Venous pulsatility index	0.116	−0.567–0.799	0.73
Plasma volume (*v*_*P*_, ×100)	−0.594	−0.987 to −0.202	0.0037
Arterial pulsatility index	0.119	−0.127–0.365	0.34

Tissue‐specific predictors include permeability surface area product (PS), cerebrovascular reactivity (CVR), blood plasma volume (*v*_*P*_) in normal‐appearing white matter (NAWM) or WMH using separate models, venous pulsatility index and adjusted for key vascular risk factors. All results are reported as unstandardized *B* value, 95% confidence interval (95% CI), and *p* value. In separate models, *v*_*P*_ was substituted for permeability surface area product (PS), and arterial pulsatility index for venous pulsatility index as the variables were derived from the same data source and to avoid overspecifying the model.

CADASIL, cerebral autosomal dominant arteriopathy with subcortical infarcts and leukoencephalopathy; WMH = white matter hyperintensity.

**Figure 3:**
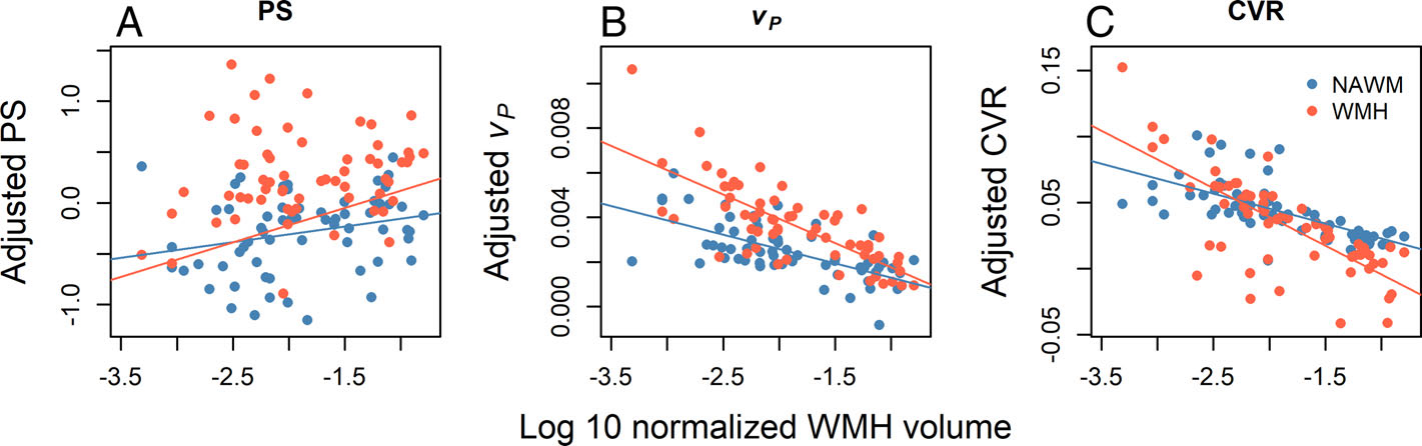
Graphs showing the interaction between vascular functions adjusted for age, smoking status, systolic blood pressure variability, tissue type (normal appearing white matter [NAWM, blue] and white matter hyperintensities (WMH; red), and the remaining vascular functions against WMH volume. (A) Permeability surface area product (PS); (B) blood plasma volume (vP)*, and (C) cerebrovascular reactivity (CVR)*. See Table 4 for coefficients. *Conventionally significant interaction (*p* ≤ 0.05) between WMH and tissue‐of‐interest (NAWM or WMH).

We found the vascular functions differed more between NAWM and WMH than between SVD subtypes (Table 4). For example, CVR did not differ between CADASIL and sporadic SVD patients (*B* = 0.0169, 95% CI −0.0247, 0.0584, *p* = 0.42), but was lower in WMH than in NAWM (*B* = −0.048, 95% CI −0.079, −0.017, *p* = 0.0033). Indeed, there were interactions between WMH volume and tissue type such that the worse the WMH volume the steeper the difference in *v*_*P*_ and CVR between NAWM and WMH (Table 4, Fig 3). Furthermore, the association with tissue damage was such that in both tissues, patients with larger WMH volumes had lower CVR (NAWM: *B* = −0.023, 95% CI −0.055, 0.010, *p* = 0.17; WMH: *B* = −0.044, 95% CI −0.077, −0.011, *p* = 0.01), lower *v*_*P*_ (NAWM: *B* = −0.00129, 95% CI −0.00250, −0.00008, *p* = 0.0037; WMH: *B* = −0.00218, 95% CI −0.00344, −0.00091, *p* = 0.0011), and a tendency to higher PS (NAWM: *B* = 0.154, 95% CI −0.551, 0.859, *p* = 0.66; WMH: *B* = 0.106, 95% CI −0.621, 0.834, *p* = 0.77).

**Table 4:** Linear Mixed Models with Tissue‐Based Vascular Functions Measured in Normal‐Appearing White Matter and White Matter Hyperintensities as Outcome Adjusting for the Remaining Vascular Functions and Key Covariates.

	Outcome	Predictor	*B*	95% CI	*p* value
(a)	PS	Intercept	0.47	−2.18–3.11	0.73
		Age	−0.0151	−0.0426–0.0124	0.28
		Smoker	−0.134	−0.618–0.350	0.58
		log_10_(WMH vol/ICV)	0.154	−0.551–0.859	0.66
		Venous pulsatility index	1.23	−0.57–3.03	0.18
		CADASIL vs sporadic	0.042	−0.858–0.943	0.93
		Systolic BP variability	6.2	−6.3–18.6	0.32
		CVR	−0.85	−4.72–3.02	0.66
		Tissue type (WMH vs NAWM)	0.46	−0.18–1.10	0.16
	log_10_(WMH vol/ICV) × tissue type		−0.047	−0.371–0.276	0.77
(b)	*v*_*P*_	Intercept	0.014	−0.430 – 0.458	0.95
		Age	0.00269	−0.00193–0.00731	0.25
		Smoker	−0.0067	−0.0874–0.074	0.87
		log_10_(WMH vol/ICV)	−0.129	−0.250 to −0.008	0.037
		Venous pulsatility index	−0.180	−0.484–0.124	0.24
		CADASIL vs sporadic	0.018	−0.133–0.168	0.81
		Systolic BP variability	2.34	0.26–4.43	0.028
		PS (×10,000)	−0.0050	−0.0416–0.0317	0.79
		CVR	0.89	0.12–1.66	0.024
		Tissue type (WMH vs NAWM)	−0.042	−0.197–0.113	0.59
	log_10_(WMH vol/ICV) × tissue type		−0.089	−0.167 to −0.011	0.025
(c)	CVR	Intercept	0.05	−0.07–0.17	0.40
		Age	−0.0011	−0.0023–0.0002	0.096
		Smoker	−0.006	−0.029–0.016	0.57
		log_10_(WMH vol/ICV)	−0.023	−0.055–0.010	0.17
		Venous pulsatility index	0.077	−0.006–0.160	0.068
		CADASIL vs Sporadic	0.0169	−0.0247–0.0584	0.42
		Systolic BP variability	−0.5	−1.0–0.1	0.10
		PS (×10,000)	−0.0015	−0.0106–0.0075	0.73
		Tissue type (WMH vs NAWM)	−0.048	−0.079 to −0.017	0.0033
	log_10_(WMH vol/ICV) × tissue type		−0.021	−0.037 to −0.005	0.011

All results are reported as unstandardized *B* value, 95% confidence interval (95% CI), and *p* value. In separate models, and arterial pulsatility index for venous pulsatility index as the variables were derived from the same data source and to avoid overspecifying the model.

CVR = cerebrovascular reactivity; ICV = intracranial volume; vol = volume.

### Relationships Between Vascular Functions

We found only nominal associations between most vascular function metrics. PS tended to be higher in patients with lower CVR (*B* = −0.85, 95% CI −4.72, 3.02, *p* = 0.66), higher venous pulsatility (*B* = 1.23, 95% CI −0.57, 3.03, *p* = 0.18), and lower arterial pulsatility (*B* = −0.084, 95% −0.780, 0.612, *p* = 0.81) (Table 4 and S7). *v*_*P*_ tended to be higher in patients with lower PS (*B* = −0.0050, 95% CI −0.0416, 0.0317, *p* = 0.79), lower venous pulsatility (*B* = −0.180, 95% CI −0.484, 0.00124, *p* = 0.24), lower arterial pulsatility (*B* = −0.094, 95% −0.207, 0.018, *p* = 0.099), and higher CVR (*B* = 0.89, 95% CI 0.12, 1.66, *p* = 0.024). CVR tended to be higher in patients with higher venous (*B* = 0.077, 95% CI −0.006, 0.160, *p* = 0.068) and lower arterial (*B* = −0.0007, 95% CI −0.0335, 0.0321, *p* = 0.97) pulsatility.

### Main Sources of Variability in SVD

**Figure 4:**
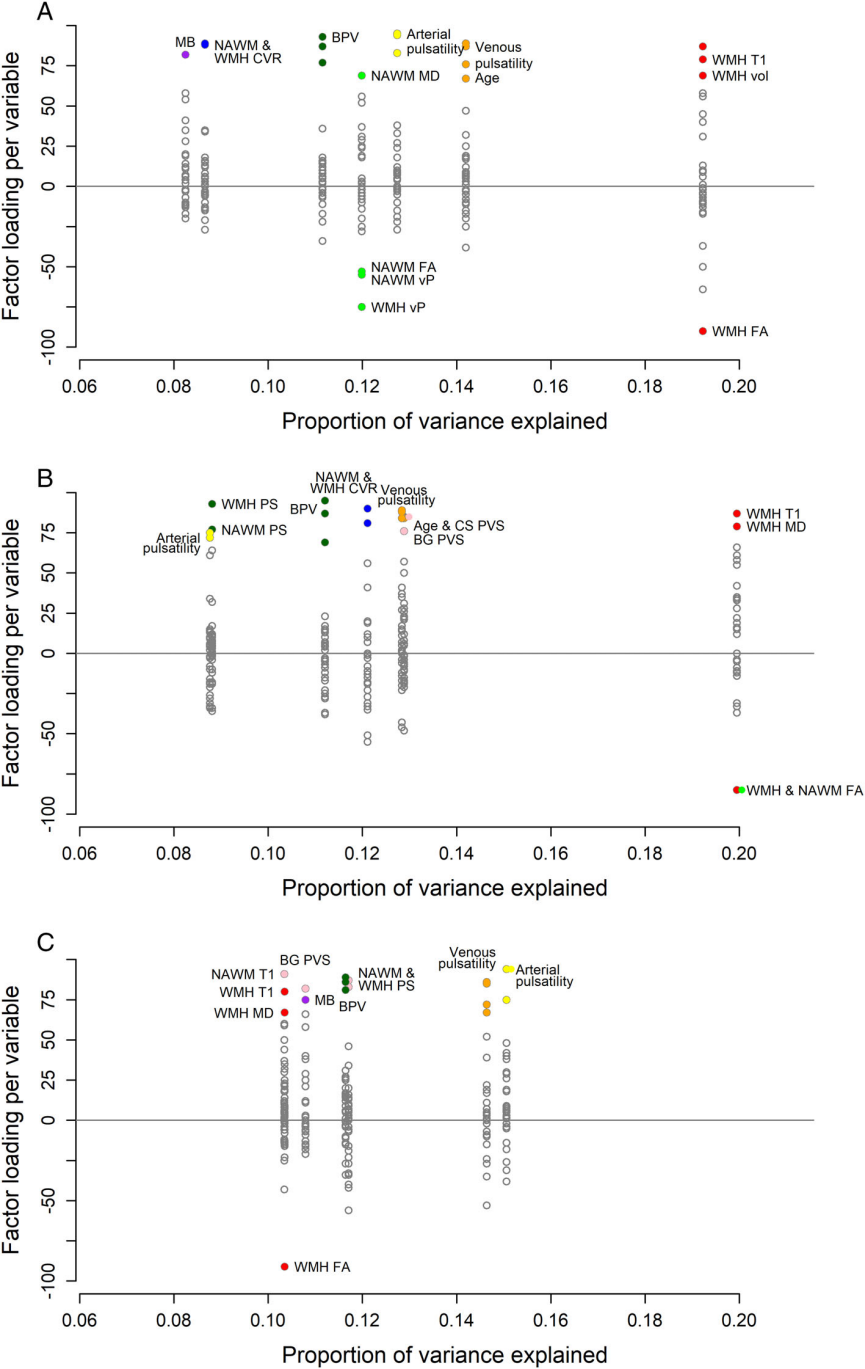
Principal component analysis. Factor loadings for each variable (*y*‐axis) versus variance in the data explained by each component for (A) all patients together, (B) cerebral autosomal dominant arteriopathy with subcortical infarcts and leukoencephalopathy only, and (C) sporadic small vessel diseases only (*x*‐axis). The labels describe the variables included in each factor. Color of variables reflects their original component in the “all patient” principal components analysis. BG = basal ganglia; BPV, blood pressure variability; CS = centrum semiovale; FA = fractional anisotropy; MB = microbleed; MD = mean diffusivity; NAWM = normal appearing white matter; PS = blood–brain barrier leakage (permeability surface area); PVS = perivascular space score; *v*_*P*_ = blood plasma volume; WMH = white matter hyperintensity volume.

In the PCA including all patients (Fig 4a), the highest proportion of variance was explained by a factor representing WMH volume, WMH *T*_*1*_, FA, and MD. The remaining factors, in decreasing order of variance explained, were venous pulsatility and age; arterial pulsatility; WMH *v*_*P*_, NAWM FA, MD, and *v*_*P*_; BPV; WMH and NAWM CVR; and number of microbleeds.

The factor order and explained variance differed between disease subtypes. In patients with CADASIL (Fig 4b), a factor representing NAWM FA, WMH *T*_1_, FA, and MD explained the most variance, followed by age; PVS score; venous pulsatility; NAWM and WMH CVR; BPV; WMH and NAWM PS; and arterial pulsatility. In patients with sporadic SVD (Fig 4c), a factor representing arterial pulsatility explained most variance, followed by venous pulsatility; WMH and NAWM PS; BPV; number of microbleeds; WMH FA, MD, and *T*_1_; and NAWM *T*_1_. In general, FA/MD explained less variance in sporadic SVD patients than in either the whole group or CADASIL patients. Of the vascular function measures, more variance was explained by CVR in CADASIL patients and PS in sporadic SVD patients, whereas venous and/or arterial pulsatility explained a similar amount in both subtypes.

## Discussion

In this international, multicenter study, thought to be the first such, we concurrently assessed 3 key vascular functions—BBB leakage, blood pulsatility and CVR, and structural brain damage—in patients with sporadic and genetic SVD. We found more severe SVD was associated with worse vascular function in sporadic SVD and CADASIL patients. Tissue type (ie, NAWM/WMH) generally more strongly predicted vascular function differences than SVD subtype. Despite SVD subtypes differing in clinical presentation, disease severity and presumed pathogenesis underlying vascular functions and tissue structural changes were similar. However, the 3 vascular functions were generally not closely interrelated, suggesting that although all 3 functions may contribute to SVD pathogenesis, they may do so at different stages in lesion development.^32^, ^33^ We also showed the feasibility of assessing 3 complex vascular functions concurrently in a multicenter MRI study, and provided a robust protocol for use as intermediary outcomes in future clinical trials testing potential SVD treatments.^18^

### Associations Between Disease Burden and Vascular Function Metrics

Consistent with previous single‐center studies testing individual vascular functions in 1 SVD subtype (not all co‐variate adjusted), we found patients with higher WMH burden tended to have higher BBB leakage,^10^, ^34^ and arterial and venous pulsatility,^9^ but lower *v*_*P*_ ^34^ and CVR^12^, ^35^ in NAWM and WMH. Mirroring our findings, several studies reported higher BBB leakage in patients with more severe WMH^6^, ^10^; higher WMH burden has generally been linked to higher PS and lower *v*_*P*_ in sporadic SVD.^6^, ^34^ Higher arterial and venous PC‐MRI pulsatility was associated with higher WMH burden in sporadic SVD,^9^, ^36^ and patients with CADASIL had higher arterial transcranial Doppler pulsatility than healthy controls.^37^ Lower CVR was linked to higher WMH burden in both sporadic SVD^12^ and CADASIL patients,^38^ consistent with recent 7‐T studies.^39^, ^40^

### Associations Between SVD Subtype and Vascular Functions

Despite higher WMH severity in CADASIL patients, we found negligible evidence that vascular functions differed between SVD subtypes adjusting for key covariates, disease severity, and tissue type. Few previous studies included sporadic SVD and CADASIL patients. One found higher BBB leakage than healthy controls in patients with sporadic SVD patients, but not CADASIL patients (all n = 20)^11^; but did not report *v*_*P*_, correct for scanner drift, and controls were older, despite age being associated with higher BBB leakage.^41^ Although higher BBB leakage was not found in transgenic CADASIL mouse models,^42^ patient cerebrospinal fluid/serum albumin ratio was elevated (n = 89).^43^ Basal ganglia regions with perivascular iron accumulation had higher BBB leakage in symptomatic/asymptomatic CADASIL patients (n = 10/11) than controls.^44^ In CADASIL patients, higher fibrinogen extravasation was reported on histopathology in WMH around enlarged PVS and lacunes,^42^ a sign of BBB leakage. In unadjusted analysis, no CVR differences were found between patients with CADASIL (n = 10) versus sporadic SVD with moderate/severe WMH (n = 20/12).^45^

PS differences between CADASIL and sporadic SVD patients may suggest PS increases occur earlier, whereas CVR reduction could become the dominant function in established severe disease. However, underlying *v*_*P*_ and vascular surface area differences may contribute.^34^ Together with reported regional differences in WMH characteristics,^46^, ^47^ spatially localized analysis methods^32^ and larger samples size are needed to investigate how BBB function varies between, and within, SVD subtypes and tissues.

### Similarities and Differences between NAWM and WMH with Increasing WMH Volume

Lower CVR and *v*_*P*_ were more strongly associated with higher WMH burden in WMH than NAWM. Few studies measured WMH CVR,^8^, ^35^, ^39^ although CVR was lower in WMH than NAWM, and, in 1 study, NAWM evolved into WMH at 1 year.^13^ Although some studies found stronger associations between PS, *v*_*P*_ and disease burden in NAWM than WMH,^34^, ^48^ others reported stronger associations in WMH.^49^ As PS combines permeability and vascular surface area, microvessel density decreases complicate interpretation; for example, in damaged tissue, partly reflected here in lower *v*_*P*_ in WMH and consistent with steeper *v*_*P*_ decline with higher WMH burden in WMH than NAWM.^34^ Given microvessels are likely to be sparse in patients with severe WMH seen in CADASIL, indicated by lower *v*_*P*_, true brain microvessel permeability is likely much higher than reflected by measured PS.^50^ Several factors, including patient population, sample size, methodology, and disease stage, potentially associated with higher/lower disease burdens or more/less acute effects, may also contribute.^6^, ^34^

### Relationships Between Different Vascular Functions

We did not generally find strong interrelationships between different vascular functions, as reflected in the often broad confidence intervals, only associations between higher *v*_*P*_ and higher CVR reached conventional significance, whereas CVR trended higher with venous pulsatility. However, the directions of effects agree with our initial hypotheses; for example, patients with higher PS tended to have higher venous pulsatility and lower CVR.

Although cross‐sectional, the present findings suggest only limited overlap between different functions, as reflected in the PCA results. Therefore, each vascular function may have a complementary role, possibly differing in order of occurrence in SVD pathogenesis. We are not able to determine the time order of vascular functions in this cross‐sectional study, but considering the disease severity associations, we could speculate that BBB leakage increases early,^11^, ^32^ followed by increases in pulsatility, decline in CVR, and *v*_*P*_ as damage accumulates. Future studies should assess longitudinal associations between vascular function metrics and tissue changes.

### Principal Component Analysis

The PCA showed that although WMH volume and quantitative tissue microstructural metrics explained the most variance in all patients and CADASIL patients, arterial pulsatility explained most variance in sporadic SVD. Venous pulsatility explained similar amounts of variance in all 3 analyses; however, arterial pulsatility explained less variance in CADASIL patients. CVR explained more variance in CADASIL patients and PS in sporadic SVD patients. As CADASIL is a more extreme SVD phenotype, differences may result from more advanced disease^1^ and, potentially, exhaustion of compensatory vascular processes. However, longitudinal replication is required to draw concrete conclusions, as the subtype analyses were exploratory.

### Strengths/Limitations

The strengths of this study included concurrent assessment of multiple vascular functions in patients with two SVD subtypes, multicenter recruitment, rigorous data acquisition, processing, and quality assurance,^7^, ^9^, ^14^, ^19^, ^20^ following consensus recommendations^6^ and best practice.^8^ We reduced artifacts and tissue signal contamination while maximizing tissue inclusion for vascular functions and quantitative measures. We demonstrated the feasibility of using these complex MRI measures in a multicenter study (>90% of patients provided usable data), demonstrating they can be used as intermediary outcomes in clinical trials of interventions in SVD.^18^ We assessed relationships between variables by interpreting findings in the context of direction of effect, confidence interval breadth, and existing clinical knowledge, rather than solely *p* values.^29^, ^30^

Limitations included shortcomings of existing methods to measure vascular function in vivo. Although we used an established well‐validated approach, BOLD‐MRI only indirectly measures blood flow and cerebral blood, volume but other factors contribute to BOLD signal changes.^8^, ^51^ However, response to 6% CO_2_ reflects capillary, as well as arteriolar dilation.^52^ Susceptibility artifact and patient motion may confound accurate CVR measurement, although we were careful to minimize artifacts. DCE‐MRI measurements of BBB leakage are limited by the low‐level permeability changes and background noise, likely contributing to the broader confidence intervals in some analyses involving BBB leakage. However, DCE‐MRI remains the consensus technique for measuring subtle BBB leakage.^6^ Whereas an established technique, 2D PC‐MRI has limited field of view, and anatomical variability can make image plane positioning challenging,^9^ although we adopted a harmonized imaging protocol. For dMRI, we used an established method^27^ using all available data (Supplementary material); however, alternative, more advanced (although less widely validated) approaches exist,^53^ which could be explored in future. As a hypothesis‐generating work, we refer to the direction of effect, even where broad confidence intervals indicate limited confidence, further methodological development may help refine these estimates. The present findings also provide the first data for a multicenter analysis of 3 vascular dysfunction measures, providing a basis for meaningful sample size calculations for similar studies in future. The long imaging protocol may bias recruitment to more physically able patients. Due to recruitment practicalities, all patients with CADASIL were recruited at a single site, with no repeat scanning of the same patients, and a separate “healthy” control group was not acquired, as healthy controls do not account for medication, co‐existing conditions, and SVD prevalence in “normal” aging, adding little to the study design.^1^ However, scans were acquired on 3‐T scanners from one vendor, and sites conducted routine quality assurance volunteer and phantom assessments (Supplementary material).^14^ Inherent differences in tissue volumes between patients with sporadic SVD and CADASIL may influence results; for example, WMH volumes were generally larger and NAWM smaller in CADASIL patients than sporadic SVD patients. More diffuse and smaller clusters of tissue are more susceptible to partial volume effect, potentially influencing measurements, particularly in uneroded WMH. Although SGM and NAWM were eroded to minimize contamination, we maximized the amount of included tissue. Due to the limited sample size, we did not evaluate interaction terms for vascular function measures and SVD subtype, nor associations with sex.

Longitudinal studies are required to determine if different vascular functions predominate at different stages of disease, and how each function contributes to SVD lesions. Further translational research and histological validation is needed to better understand hemodynamic measures and their interdependencies,^50^ and whether vascular function can be improved with interventions.^18^

## Conclusion

We showed that 3 vascular function mechanisms (BBB leakage, CVR, and blood pulsatility) occur in both CADASIL and sporadic SVD patients, are all associated with WMH severity, and differ between WMH/NAWM. Associations between different vascular functions and SVD burden suggest a complex, sequential process. Despite stark differences in visible SVD burden, similar vascular functions are implicated in both SVD subtypes. Although inferences on vascular function evolution from this cross‐sectional study are limited, the association analysis and PCA may suggest differential evolution, with BBB leakage increasing early, followed by increased pulsatility, and declining CVR and *v*_*P*_ as microvascular and tissue damage accumulates. Finally, we showed that complementary sophisticated vascular functions measures can be assessed in multicenter SVD studies with minimal data loss, providing intermediary outcome measures for clinical trials and observational studies.

## Author Contributions

J.M.W., M.Di., G.J.B., and R.vO. contributed to the conception and design of the study; M.S.S., G.W.B., A.K., D.K., M.J.T., F.M.C., S.M.M., R.B., K.S., I.H., D.J.G., F.N.D., U.C., E.S., T.P., E.J., M.Du., M.I., J.S., W.H.B., and members of the SVDs@Target consortium contributed to the acquisition and analysis of data; M.S.S., M.J.T., F.M.C., and J.M.W. contributed to drafting the text or preparing the figures.

## Potential Conflicts of Interest

Nothing to report.

## Supporting information


**Data S1.** Supporting Information.

## Data Availability

The study data are available from the SVDs@target collaborative group via the corresponding author, upon reasonable request.
