# Supplementary material for: Cerebrovascular Function in Sporadic and Genetic Cerebral Small Vessel Disease
Source: Ann Neurol. 2024 Nov 18;97(3):483–98. doi: 10.1002/ana.27136 (PMC11831873; doi:10.1002/ana.27136)
Supplement: Supplementary file 1 — Data S1. Supporting Information. [file ANA-97-483-s001.docx]

**Supplementary material**

**Methods**

**Quality assurance**

**Image acquisition**

**Image processing**

**Results**

**Quality assurance**

**Figures**

**Supplemental Figure 1:** Mean signal slope by site for the dummy DCE-MRI scans by site

**Supplemental Figure 2:** Flow chart of recruitment by site and dropout.

**Supplemental Figure 3:** Panel of graphs of key imaging characteristics by SVD subtype: a) tissues volumes, b) WMH volume, c) number of lacunes and d) number of microbleeds.

**Tables**

**Supplemental Table 1:** Acquisition parameters for the MR imaging protocol

**Supplemental Table 2:** Variables included in the PCA

**Supplemental Table 3:** Summary parameters derived from the ACR phantom scans

**Supplemental Table 4:** Descriptive statistics for key demographic, medical, visual ratings and tissue volumes by site.

**Supplemental Table 5:** Descriptive statistics for the tissue and vascular dysfunction metrics by tissue type and site

**Supplemental Table 6:** Additional summary statistics for the phase contrast data by disease subtype.

**Supplemental Table 7:** Linear mixed models with arterial pulsatility replacing venous pulsatility as a predictor

**Supplemental Table 8**: SVDs@Target collaborators

**Methods**

**Quality Assurance**

Each site acquired a healthy volunteer test scan to assess the MRI protocol accuracy, scan quality, comparability and check the data transfer process.

To ensure comparability between sites, we implemented a harmonised QA programme to supplement routine local QA which included: 1) local monitoring of SNR using site specific QA protocols at least monthly; 2) phantom scans using the large or small American College of Radiologists phantom^1^ and accreditation protocol,^2^ at the beginning, during and end of the project; 3) at least 5 healthy volunteer scans without Gd-based contrast agents using the DCE-MRI protocol to monitor scanner drift over the course of the project.

For the duration of the study all sites followed a regular in-house quality assurance programme, including local routine monitoring of signal-to-ratio. To help insure comparability and data quality we also conducted phantom scans using the large or small American College of Radiologists phantom^1^ and accreditation protocol,^2^ at least at the beginning, during and end of the project. Analysis of the ACR phantom data was conducted centrally by a single rater in ImageJ (imagej.nih.gov/ij).

We also conducted several healthy volunteer scans across the course of the study to assess scanner drift using the same sequence as the DCE scan without contrast.

**Image acquisition**

During the 12-minute CVR scan we alternated between 2 minutes of medical air and 3 minutes of 6% CO_2_ in air (BOC Special Products; Guildford, United Kingdom) while patients wore a carefully fitted anaesthetic face mask attached to a unidirectional open breathing circuit (Intersurgical, Wokingham, United Kingdom).^3^ We recorded end-tidal CO_2_ (ETCO2) throughout (CD3-A AEI Technologies, Pittsburgh, PA). Patients were monitored by a physician during the *CVR* scan.

We acquired the arterial and venous flow waveforms to assess vascular pulsatility using a 2D cine phase-contrast sequence with retrospective peripheral pulse gating to acquire 32 velocity images per cardiac cycle. To measure flow waveforms through different structures we acquired images in four planes: superior to the carotid bifurcation and perpendicular to the internal carotid artery (ICA) walls to measure flow in the ICAs, vertebral arteries (VAs), and internal jugular veins (IJVs); a coronal-oblique slice intersecting the superior sagittal sinus (SSS) around 2 cm above the torcular and through the mid-point of the straight (StS) to measure flow in the SSS, StS and transverse sinuses (TS); perpendicular to the cerebral aqueduct to measure CSF flow; and an axial slice at the craniocervical junction to measure foramen magnum CSF flow.^4^

We measured quantitative *T_1_* and flip angle using the driven equilibrium single pulse observation of *T_1_* with high-speed incorporation of RF field inhomogeneities (DESPOT1-HIFI) method, acquiring two 3D inversion-recovery prepared spoiled gradient echo (IR-sGRE) sequences at different inversion times (600 and 1500 ms) and three 3D spoiled gradient echo sequences (sGRE) with different flip angles (2, 5 and 12**°**).^5^

We acquired DCE-MRI using a 3D sagittal T1-w sGRE with 32 volumes acquired at a temporal resolution of 39.6s. After acquiring three pre-contrast volumes, we intravenously injected a dose of 0.1 mmol/kg body weight gadobutrol (1 M Gadovist, Bayer AG, Leverkusen, Germany) delivered over a period of 110-130s at Edinburgh and Munich or 40-150s at Maastricht using a power injector followed by a 20ml saline flush.^6^

**Image processing**

*Regions of interest (ROIs)*

We evaluated several segmentation approaches, including manually defined ROIs using an established protocol and computational masks with different levels of erosion attempting to maximising the tissue volume included while minimising partial volume effect.

The manual approach included substantially less tissue than any of the computationally determined masks in subcortical grey matter and normal appearing white matter masks. However, the tissue volume in the manual more consistent than the computational masks, which may affect the signal-to-noise ratio and hence the accuracy of the *CVR* values. The computational approach reduced the potentially confounding effect of differing proportions of NAWM vs WMH, which was potentially particularly relevant for studying CADASIL patients.

For the computational masks we assessed erosion using the spherical and boxv kernels in fslmaths^7^ set to either 2 and 4mm. At 4mm erosion we found most of the tissue volume was lost, though minimal manual checking was required. While the spherical kernel included more tissue, contamination from cortical grey matter and around major blood vessels was substantially higher. Based on these factors we determined that the 2mm boxv kernel provided the best compromise, including substantially more tissue than either the manual or 4mm kernels and requiring relatively minimal editing.

We therefore eroded both the SGM and NAWM masks using FSL by a two voxel rim around each of the edges in the structural space, equivalent to 2 mm or 1 voxel in *CVR* space, to reduce the influence of partial volume effect.

As we noted that vessels running on the inner ventricular surface tended to contaminate the BOLD signal. We dilated a ventricle mask, derived from FSL FAST, by 5 voxels (5mm) left-right, 4 voxels (4mm) top-bottom and front-back in each direction (whether it was WM or WMH). We overlaid the resulting masks on the voxelwise *CVR* map in FSLEyes for each subject and inspected with reference to the SWI sequence to confirm the penetrating vessels had been removed, exclude blooming from large veins and venous sinuses using the *CVR* map. All masks were checked and manually edited as needed to avoid misclassification.

*dMRI*

We (S.M.M) processed the multi-shell diffusion MRI data through TractoR (version 3.3),^8^ preferred in cohorts with high disease burdens.^9, 10^ After converting to NIFTI format using divest (<https://CRAN.R-project.org/package=divest>)^11^ we corrected the data for susceptibility and eddy current artefacts as well as subject motion using FSL topup^12^ and eddy.^13^ We masked the brain using a mask generated in FSL bet^14^ and calculated the water self-diffusion tensor per voxel using iterative weighted least-squares through tensorfit.^15^ This limits the contribution of shells at higher b-values, which have lower signal-to-noise ratio, where the assumption of linearity is violated while using all available data. Using the eigenvalues, we derived maps of fractional anisotropy (FA) and mean diffusivity (MD). We applied boundary-based registration using the total white matter mask (normal appearing and WMH) to register the non-diffusion weighted image, S0, to the T2-w space to minimise the influence of distortion artefacts.^16^ We inverted the matrices using convert_xfm to transform the tissue masks into the diffusion MRI space and extracted the median FA and MD values of each region using fslstats.^7^

*Quantitative T_1_*

We (M.J.T) calculated pre-contrast quantitative *T_1_* _­_maps corrected for $B_{1}^{+}$ error as previously described using in-house MATLAB scripts (<https://github.com/mjt320/HIFI>).^6, 17^

*DCE-MRI*

We spatially realigned the brain extracted^14^ DCE-MRI images using SPM12.^18^ Per consensus guidelines^19^ we estimated a vascular input function by manually selecting five voxels in the SSS and taking the mean value at each time point using an established method.^6^ We registered the T2-w scan to the mean pre-contrast image using FSL FLIRT,^20^ and transformed the tissue masks into the DCE space. We (M.J.T) calculated the signal enhancement for each volume relative to the mean pre-contrast signal before estimating the Patlak tracer kinetic parameters, permeability surface area (*PS*) and blood plasma volume (*v_P_*), assuming the no-exchange limit for water transport across the BBB and corrected for $B_{1}^{+}$ error.^6^ As we found evidence of a possible site-specific scanner drift bias in healthy volunteer scans without Gd-based contrast injection (Supplementary Figure 1), consistent with previous work,^21^ we applied a site-dependent correction factor to offset the mean drift calculated from the control data.

*Phase contrast*

We (M.S.S.) manually delineated regions of interest for the internal carotid arteries, internal jugular veins, straight, tranverse and venous sinuses, and subarachnoid CSF in FSLEyes^22^ using an established protocol.^4^ Using carefully positioned background ROIs placed near to the vessels in regions with no visible flow we corrected for background phase error.^23^ For each vessel we summed the total flow across the cardiac cycle and calculated the pulsatility index based on an adapted version of Gosling’s equation.^4^ We calculated subarachnoid CSF stroke volume by averaging the absolute flow volume in caudal and cranial directions.^4, 24^

*CVR*

We converted the BOLD data to NIFTI format,^11^ discarded the first 7 volumes as dummy scans and realigned the remaining volumes to the mean using the two-pass procedure implemented in SPM12 (<https://www.fil.ion.ucl.ac.uk/spm/>).^18^ Using in-house MATLAB code, we identified the CO_2_ peaks, converted the recordings to ETCO_2_, and resampled to match the temporal resolution of the BOLD data.^3^ We registered the mean BOLD image to the T2-w space using rigid-body registration with FSL FLIRT^20^ and inverted the transformation matrix to register the tissue masks into the BOLD space. For each mask, we (M.S.S) performed multiple linear regression between the mean percentage signal change relative to baseline (dependent variable), and the time-shifted ETCO_2_ profile and volume number (correcting for drift) to calculate the *CVR* magnitude. To account for delay,^25^ we evaluated the model at a range of time-shifts and selected the model with the lowest sum-of-square residuals. We added 4 s to account for the transit time of sampled gases from the patient to the monitor based on previous work^3^ and after measuring the offset at each site.

**Supplementary Results**

**Quality assurance**

All ACR phantom scans met or exceeded the minimum recommendations. For summary statistics for the ACR phantom measurements see Supplemental Table 3.

In total 26 healthy volunteers were recruited (n=16/5/5 at Edinburgh/Maastricht/Munich respectively). While stability was reasonable at all three scanners on average there was a slight negative trend in the dummy scans from Munich (Supplemental Figure 1).

**Supplemental Figures**

**Supplemental Figure 1**: Mean signal slope by site for the dummy DCE-MRI scans (ED=Edinburgh, MU=Munich, MA=Maastricht).


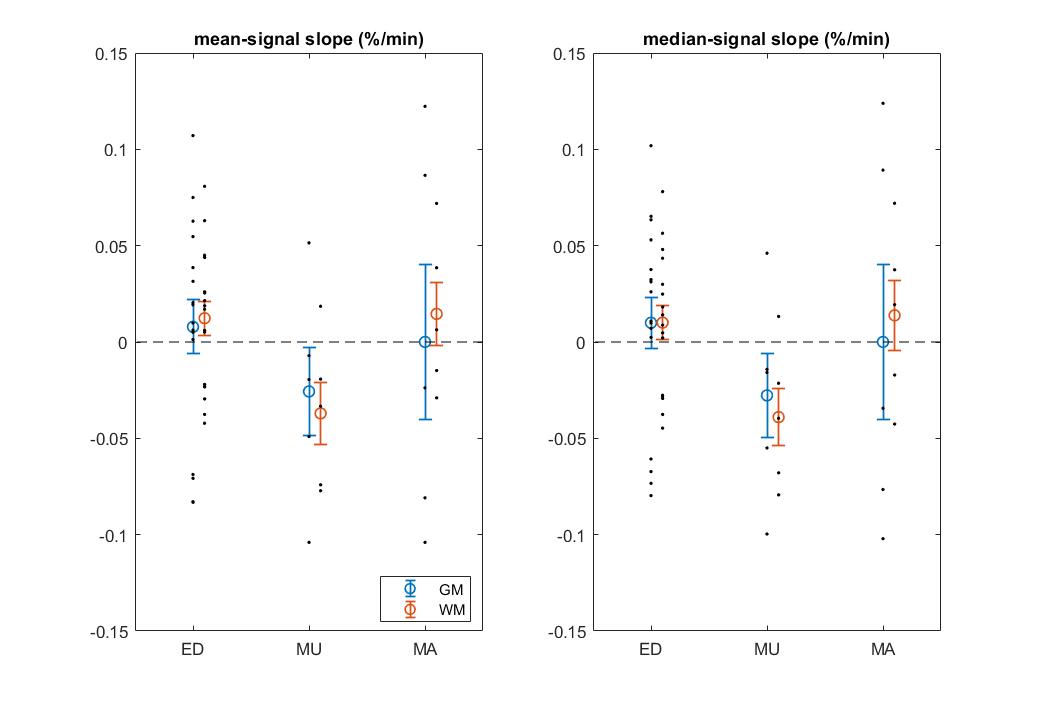


**Supplementary Figure 2:** Flow chart of recruitment by site and dropout.


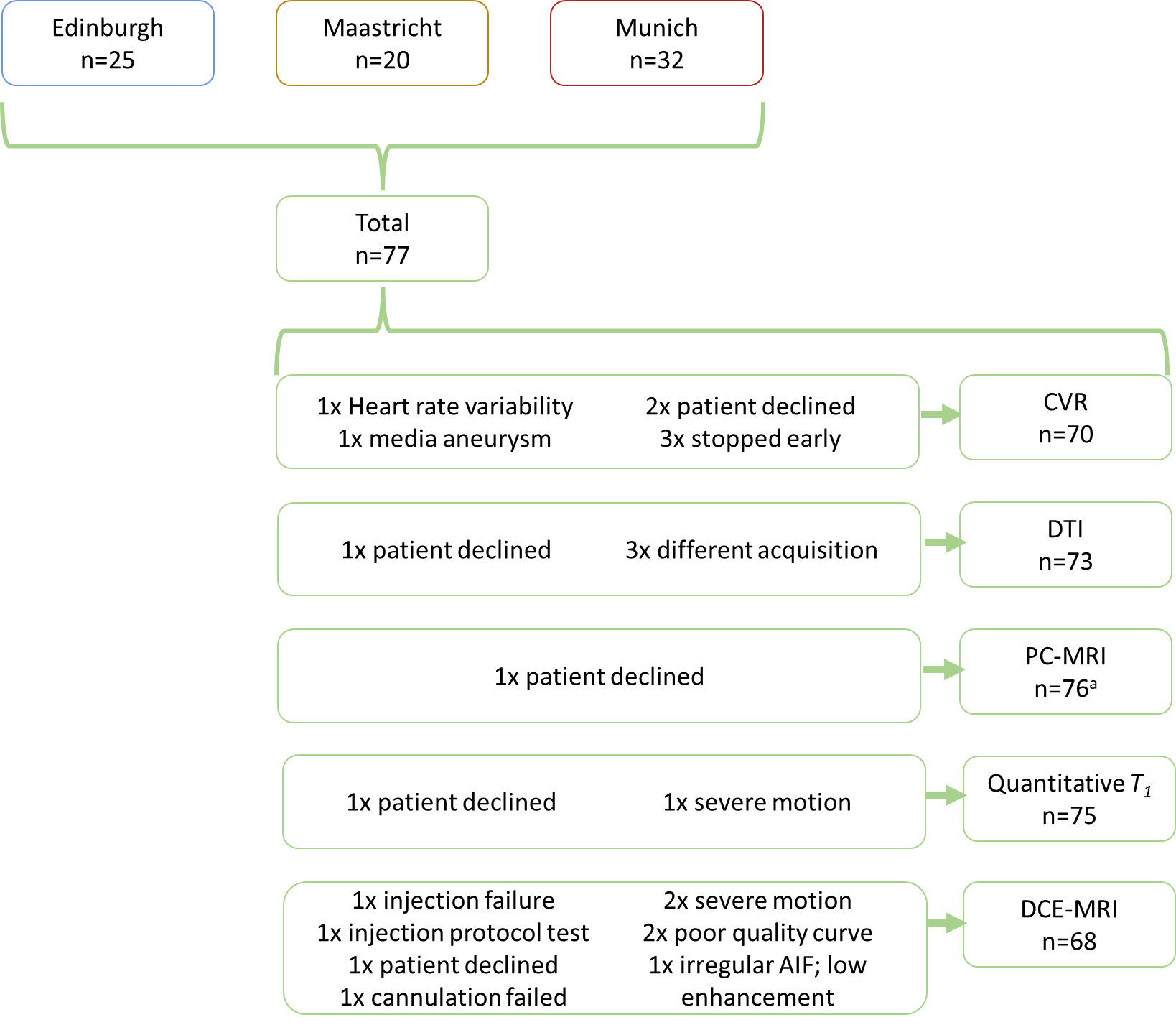


**Supplementary Figure 3:** Panel of graphs of key demographic and imaging characteristics by SVD subtype: a) age, b) hypertension diagnosis, c) b lood pressure (BP) variability, d) smoking status and e) total PVS score.


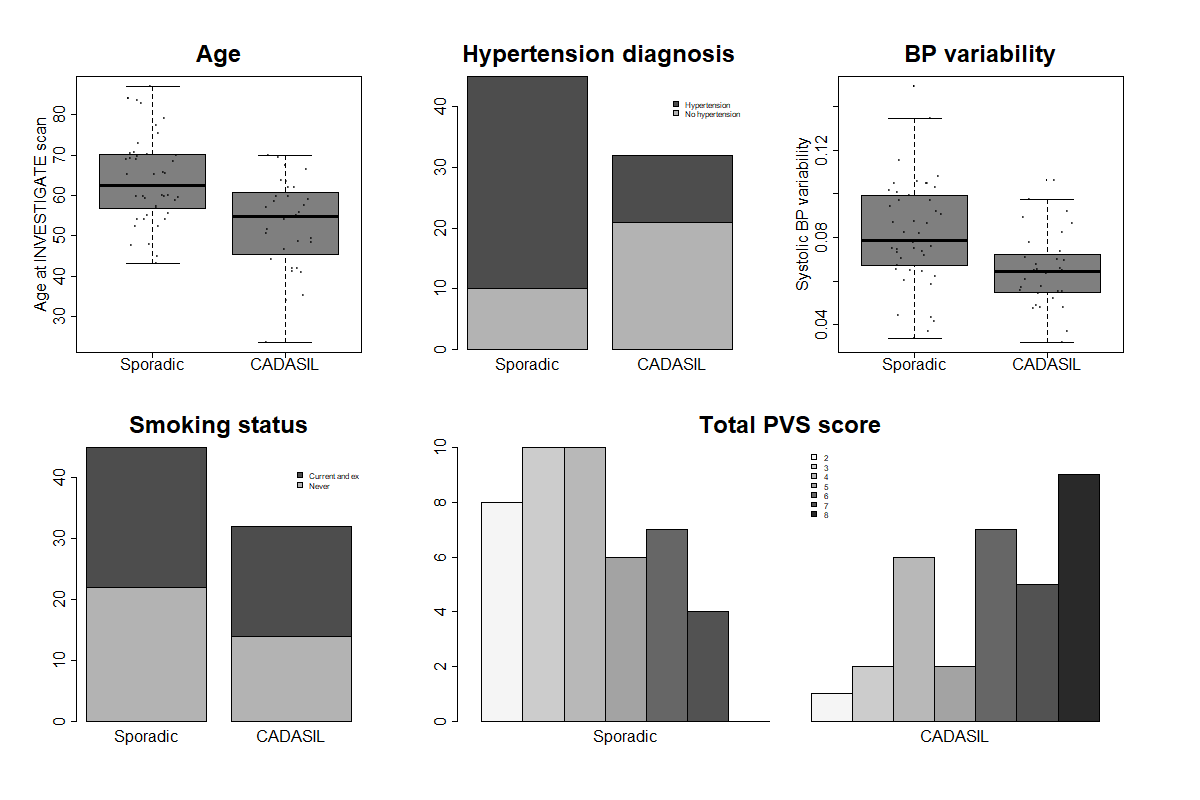


(b)

(e)

(d)

(c)

(a)

**Supplemental Tables**

**Supplemental Table 1**: Acquisition parameters for the imaging protocol. Voxel size in mm. TR/TE/TI all in milliseconds. Acquisition time in minutes and seconds. Flip angle in degrees. *CVR* =Cerebrovascular reactivity; MPRAGE =Magnetisation-prepared rapid acquisition with gradient echo; FLAIR =Fluid-attenuated inversion recovery; PD =Proton density; SPACE =Sampling perfection with application-optimised contrast using different flip-angle evolution; [SWI](https://www.sciencedirect.com/topics/medicine-and-dentistry/susceptibility-weighted-imaging) =Susceptibility weighted imaging; dMRI =Diffusion magnetic resonance imaging; TOF =Time-of-flight; PC =Phase-contrast; SACSF=Subarachnoid [cerebrospinal fluid](https://www.sciencedirect.com/topics/medicine-and-dentistry/cerebrospinal-fluid); IR =Inversion recovery; sGRE =Spoiled gradient recalled echo; TI =Inversion time; FA =Flip angle; DCE =Dynamic contrast-enhanced; TR =Repetition time; TE =Echo time; R=parallel imaging acceleration factor; MB=multiband acceleration factor; NA = number of averages.

|  | ***CVR*** | **T1-w** | **FLAIR** | **PD** | | **T2-w** | **SWI** | **dMRI** |
| --- | --- | --- | --- | --- | --- | --- | --- | --- |
| **Sequence** | **2D GE-EPI** | **MPRAGE (3D IR-sGRE)** | **SPACE (3D RARE)** | | **3D sGRE** | **SPACE (3D RARE)** | **3D sGRE** | **2D GE-EPI** |
| **Voxel size (mm)** | 2.5x2.5x2.5 | 1.0x1.0x1.0 | 1.0x1.0x1.0 | 1.0x1.0x1.0 | | 0.9x0.9x0.9 | 0.6x0.6x3.0 | 2.0x2.0x2.0 |
| **TR (ms)** | 3000 | 2500 | 5000 | 6.04 | | 3200 | 28 | 4300 |
| **TE (ms)** | 30.0 | 4.37 | 388 | 2.44 | | 408 | 20 | 74.0 |
| **TI** | - | 1100 | 1800 | - | | - | - | - |
| **Flip Angle (°)** | 90 | 7 | - | 2.0 | | - | 9 | - |
| **Acquisition Time (mm:ss)** | 12:30 | 3:45 | 5:57 | 1:57 | | 3:42 | 4.02 | 11:16 |
| **Other** | *R*=2 | *R*=3 | *R=3* | *R*=3 | | *R*=2x2 | *R*=2 | *R*=2, *MB*=2,  15 × b = 0 s/mm2, 3 × b = 200 s/mm2, 6 × b = 500 s/mm2, 64 × b = 1000 s/mm2, 64 × b = 2000 s/mm2  (3 x b0 acquired with reversed phase encoding) |

|  | **MRA** | **Flow** | | | **quantitative *T*_1_** | | | **DCE-MRI** |
| --- | --- | --- | --- | --- | --- | --- | --- | --- |
| **Sequence** | **TOF** | **2D PC (carotids)** | **2D PC (SACSF)** | **2D PC (sinus)** | **3D IR-sGRE**  **(TI = 600 ms)** | **3D IR-sGRE**  **(TI = 1500 ms)** | **3D sGRE**  **(FA = 2°,5°, 12°)** | ***T*1w 3D sGRE** |
| **Voxel size (mm)** | 0.5x0.7x1.6 | 1.0x1.0x5.0 | 0.8x0.8x5.0 | 0.7x0.7x5.0 | 1.2x1.2x1.2 | 1.2x1.2x1.2 | 1.2x1.2x1.2 | 2x2x2 |
| **TR (ms)** | 20.0 | 19.60 | 25.18 | 21.70 | 1040 | 1940 | 5.4 | 3.44 |
| **TE (ms)** | 3.51 | 5.82 | 8.45 | 6.59 | 1.82 | 1.82 | 1.82 | 1.68 |
| **TI** | - | - | - | - | 600 | 1500 | - | - |
| **Flip Angle (°)** | 20 | 12 | 12 | 12 | 5 | 5 | 2, 5, 12 | 15 |
| **Acquisition Time (mm:ss)** | 2:45 | c.1:39 | c.1:55 | c.2:11 | 1:55 | 3:35 | 1:36 x 3 | 21:08 |
| **Other** |  | *R*=2, NA = 2  venc =70 cm s^-1^ | *R*=2  venc = 6 cm s^-1^ | *R*=2  venc = 50 cm s^-1^ | *R*=2 | *R*=2 | *R*=2 | 32 volumes |

**Supplemental Table 2**: Variables included in the Principal Component Analyses

| Source | Variables |
| --- | --- |
| Demographics | Age (years) |
| Blood pressure | Systolic, diastolic and mean arterial pressure blood pressure variability (unitless) |
| Visual score | Number of microbleeds, lacunes, Fazekas score, PVS in basal ganglia and centrum semiovale |
| Volumes | WMH volume (ml) |
| Phase contrast | Arterial, internal carotid, vertebral, internal jugular vein, transverse sinus, superior sagittal sinus, and straight sinus pulsatility index (unitless); Subarachnoid cerebrospinal fluid stroke volume (ml) |
| DCE-MRI | *PS* (10^-4^ min^-1^) and *v_P_* (10^-2^) in normal appearing white matter and WMH |
| *CVR* | *CVR* magnitude (%/mmHg) and delay (s) in normal appearing white matter and WMH |
| dMRI | Fractional anisotropy and mean diffusivity (10^-3^ mm^2^/s) in normal appearing white matter and WMH |
| Quantitative *T_1_* | *T_1_* (s^-1^) in normal appearing white matter and WMH |

**Supplemental Table 3**: Summary parameters derived from the ACR phantom scans for the three main scanners used in the study

|  | **Edinburgh** | **Maastricht** | **Munich** | **ACR recommendations** |
| --- | --- | --- | --- | --- |
| **ACR phantom** | **Large** | **Large** | **Small** | **Large/Small** |
| Geometric accuracy % (mm) | 0.12±0.36  (0.22±0.68) | 0.46±0.27  (0.88±0.52) | 0.23±0.87 (0.23±0.87) | ≤1.05 (2) / ≤2 (2) |
| Slice thickness % (mm) | 8.54±3.49  (0.46±0.18) | 9.77±0.39  (0.49±0.23) | 1.60±5.83 (0.08±0.29) | ≤14 (0.7) / ≤14 (0.7) |
| Slice position (mm) | 1.92±1.00 | 2.29±0.92 | 1.14±0.17 | ≤5mm / ≤5mm |
| Percentage ghosting (%) | 0.29±0.15 | 0.63±0.16 | 0.352±0.01 | ≤2.5% |

**Supplemental Table 4**: Descriptive statistics covering demographics, blood pressure, visual ratings and structural brain volumes. All values reported as number (percentage) for categorical, mean ± standard deviation (range) for normally distributed numeric variables and median (inter-quartile range) otherwise.

|  | **All patients** | **Edinburgh** | **Maastricht** | **Munich** | **Inter-site differences** |  |
| --- | --- | --- | --- | --- | --- | --- |
| **Demographics** | | | | |  |  |
| Total n | 77 (100.0) | 25 (100.0) | 20 (100.0) | 32 (100.0) | – | |
| Male/Female n (%) | 42/35 (54.5,45.5) | 13/12 (52.0/48.0) | 13/7 (65.0/35.0) | 16/16 (50.0/50.0) | χ^2^=1.21, p=0.55 | |
| Age (years) | 59.5± 12.3  (23.6 – 87.0) | 68.3 ± 11.3  (44.8 – 87.0) | 59.1 ± 8.3  (43.0 – 70.7) | 52.9 ±11.1  (23.6 – 70.0) | F=15.1, p<0.0001 | |
| Diabetes | 10 (13.0) | 6 (24.0) | 3 (15.0) | 1 (3.1) | p=0.067 | |
| Hypertension | 46 (59.7) | 20 (80.0) | 15 (75.0) | 11 (34.4) | χ^2^=14.8, p=0.0006 | |
| Hyperlipidaemia | 46 (59.7) | 20 (80.0) | 13 (65.0) | 13 (40.6) | χ^2^=9.36, p=0.0093 | |
| Current & Ex- smoker | 41 (53.3) | 10 (40.0) | 13 (65.0) | 18 (56.3) | p=0.43 | |
| Does use alcohol | 55 (71.4) | 17 (68.0) | 14 (70.0) | 24 (75.0) | χ^2^=0.364, p=0.83 | |
| Alcohol units /week | 1 (0 – 5) | 5 (0 – 10) | 1.5 (0 – 3) | 1 (0.5 – 2.5) | H=2.82, p=0.24 | |
| **Blood pressure** | | | | | | |
| Pre-CVR systolic (mmHg) | 143.6 ± 24.3  (90.0 – 200.0) | 159.0 ± 27.0  (90.0– 200.0) | 153.6 ± 17.4  (130.0 – 199.0) | 127.7 ± 13.9  (110.0 – 160.0) | F=19.7, p<0.0001 | |
| Pre-CVR diastolic (mmHg) | 82.2±12.3  (50.0 – 110.0) | 83.5 ± 14.0  (50.0 – 107.0) | 91.0 ± 9.4  (76.0 – 110.0) | 76.7 ± 9.5  (60.0 – 90.0) | F=9.45, p=0.0002 | |
| Mean 24-hour systolic (mmHg) | 125 ± 12.3  (100.7 – 157.1) | 130.7 ± 11.1  (106.1 – 149.2) | 129.7 ± 12.4  (105.7 – 157.1) | 117.5 ± 9.1  (100.7 – 139.9) | F=13.3, p<0.0001 | |
| Mean 24-hour diastolic (mmHg) | 80.4 ± 9.5  (60.6 – 105.4) | 80.8 ± 8.5  (62.4 – 93.8) | 85.8 ± 9.4  (73.6 – 105.4) | 76.7 ± 8.9  (60.6 – 90.2) | F=6.54, p=0.0024 | |
| Systolic BPV (unitless) | 0.0750 ± 0.0229  (0.0321 – 0.1493) | 0.0804 ± 0.0242  (0.0340 – 0.1347) | 0.0842 ± 0.0244  (0.0432 – 0.1493) | 0.0651 ± 0.0169  (0.0321 – 0.1063) | F=6.07, p=0.0036 | |
| Diastolic BPV (unitless) | 0.0801 ± 0.0235  (0.0362 – 0.1444) | 0.0806 ± 0.0195  (0.0514 – 0.1215) | 0.0801 ± 0.0241  (0.0488 – 0.1444) | 0.0796 ± 0.0266  (0.0362 – 0.1400) | F=0.0131, p=0.99 | |
| **Visual ratings** | | | | | | |
| Deep Fazekas | 2 (1 – 3) | 2 (1 – 2) | 1 (1 – 2) | 3 (2.5 – 3) | χ^2^=38.5, p<0.0001 | |
| Periventricular Fazekas | 2 (1 – 3) | 2 (1 – 2) | 2 (1 – 2) | 3 (3 – 3) | χ^2^=35.1, p<0.0001 | |
| Total Fazekas | 4 (3 – 6) | 3 (2 – 4) | 3 (2 – 4) | 6 (5 – 6) | χ^2^=41.5, p<0.0001 | |
| Basal ganglia PVS score | 2 (1 – 3) | 2 (1 – 2) | 2 (1 – 3) | 3 (2 – 4) | χ^2^=13.7, p=0.0010 | |
| Centrum semiovale PVS score | 3 (2 – 3) | 2 (2 – 2) | 3 (1 – 3) | 3 (2 – 4) | χ^2^=16.6, p=0.0002 | |
| Total PVS | 5 (3 – 6) | 4 (3 – 4) | 5 (2 – 6) | 6 (4 – 8) | χ^2^=18.8, p<0.0001 | |
| No. of lacunes | 3 (0 – 7) | 2 (0 – 4) | 1 (0 – 3) | 5.5 (1 – 9) | χ^2^=9.49, p=0.0087 | |
| No. of microbleeds | 0 (0 – 4) | 0 (0 – 1) | 0 (0 – 2.5) | 1.5 ( 0 – 8) | χ^2^=7.20, p=0.027 | |
| Deep atrophy score | 3 (3 – 4) | 3 (3 – 4) | 4 (2.5 – 5) | 3 (2 – 3) | χ^2^=6.97, p=0.031 | |
| Superficial atrophy score | 3 (3 – 4) | 3 (3 – 4) | 4 (2.5 – 5) | 3 (2 – 4) | χ^2^=2.89, p=0.24 | |
| **Structural MRI volumes (ml)** | | | | | | |
| Intracranial volume | 1413.03 ±133.37  (1142.07 – 1878.32) | 1402.61 ± 155.73  (1216.02 – 1878.32) | 1454.1 ±144.85  (1180.12 – 1731.07) | 1395.51 ±102.39  (1142.07 – 1603.28) | F=1.31, p=0.28 | |
| Brain volume | 1104.92 ±101.29  (881.47 – 1360.2) | 1071.19 ±110.84  (881.47 – 1307.42) | 1103.61 ±105.8  (925.87 – 1305.02) | 1132.08 ±84.26  (963.88 – 1360.2) | F=2.65, p=0.078 | |
| CSF volume | 302.38 ±74.54  (172.04 – 609.08) | 326.28 ±79.43  (219.21 – 609.08) | 344.44 ±59.9  (233.58 – 450.62) | 257.42 ±53.37  (172.04 – 362.92) | F=13.7, p<0.0001 | |
| GM volume | 530.64 ±51.39  (411.65 – 679.04) | 529.88 ±57.57  (421.61 – 679.04) | 543.66 ±47.09  (468.75 – 632.18) | 523.09 ±48.80  (411.65 – 629.02) | F=1.00, p=0.38 | |
| NAWM volume | 532.76 ±57.96  (421.07 – 701.99) | 530.75 ±57.19  (432.54 – 649.13) | 546.01 ±54.23  (446.86 – 655.42) | 526.05 ±61.14  (421.07 – 701.99) | F=0.747, p=0.48 | |
| WMH volume | 41.46 ±52.61  (0.64 – 220.75) | 10.36 ±8.56  (1.30 – 34.58) | 13.95 ±25.87  (0.64 – 119.92) | 82.94 ±57.22  (3.75 – 220.75) | F=50.4­^1^, p<0.0001 | |

^1^ Due to a lack of Normality in WMH volumes, the ANOVA test used log base 10 WMH volume normalised to intracranial volume instead.

**Supplemental Table 5**: Summary statistics for the dynamic imaging, quantitative *T_1_* and diffusion imaging metrics. All values reported as mean ± standard deviation (range).

|  | All patients | Edinburgh | Maastricht | Munich | Inter-site differences |
| --- | --- | --- | --- | --- | --- |
|  |  |  |  |  |  |
| **Mean diffusivity (*MD*, 10^-3^ mm^2^/s)** | | | | | |
| Subcortical grey matter | 0.70 ± 0.08  (0.54 – 1.01) | 0.66 ± 0.04  (0.63 – 0.80) | 0.66 ± 0.03  (0.62 – 0.73) | 0.74 ± 0.09  (0.54 – 1.01) | F=12.2, p<0.0001 |
| Normal-appearing white matter | 0.65 ± 0.03  (0.58 – 0.70) | 0.62 ± 0.02  (0.58 – 0.67) | 0.65 ± 0.03  (0.61 – 0.70) | 0.66 ± 0.03  (0.60 – 0.70) | F=16.9, p<0.0001 |
| White matter hyperintensity | 0.95 ± 0.11  (0.69 – 1.20) | 0.88 ± 0.09  (0.69 – 1.11) | 0.91 ± 0.09  (0.82 – 1.16) | 1.02 ± 0.09  (0.87 – 1.20) | F=19.4, p<0.0001 |
| **Fractional anisotropy (*FA*)** | | | | | |
| Subcortical grey matter | 0.24 ± 0.03  (0.16 – 0.32) | 0.25 ± 0.02  (0.20 – 0.28) | 0.26 ± 0.02  (0.23 – 0.32) | 0.22 ± 0.03  (0.16 – 0.28) | F=12.3, p<0.0001 |
| Normal-appearing white matter | 0.48 ± 0.03  (0.37 – 0.54) | 0.51 ± 0.02  (0.47 – 0.54) | 0.49 ± 0.02  (0.45 to 0.54) | 0.46 ± 0.03  (0.37 – 0.51) | F=22.1, p<0.0001 |
| White matter hyperintensity | 0.31 ± 0.06  (0.20 – 0.42) | 0.34 ± 0.04  (0.27 – 0.41) | 0.34 ± 0.05  (0.24 to 0.42) | 0.27 ± 0.04  (0.20 – 0.41) | F=25.7, p<0.0001 |
| ***T_1_* (s)** | | | | | |
| Subcortical grey matter | 1.26 ± 0.07  (1.15 – 1.53) | 1.26 ± 0.04  (1.19 – 1.36) | 1.23 ± 0.04  (1.19 – 1.32) | 1.28 ± 0.08  (1.15 – 1.53) | F=3.36, p=0.040 |
| Normal-appearing white matter | 0.94 ± 0.04  (0.87 – 1.05) | 0.94 ± 0.03  (0.88 – 1.03) | 0.94 ± 0.04  (0.88 – 1.04) | 0.95 ± 0.04  (0.87 – 1.05) | F=0.86, p=0.43 |
| White matter hyperintensity | 1.35 ± 0.11  (1.14 – 1.75) | 1.33 ± 0.12  (1.14 – 1.67) | 1.34 ± 0.13  (1.20 – 1.75) | 1.38 ± 0.10  (1.22 – 1.59) | F=2.10, p=0.13 |
| **Permeability surface area (*PS*, 10^-4^ min^-1^)** | | | | | |
| Subcortical grey matter | 0.90 ± 1.19  (-3.52 – 4.56) | 0.90 ± 1.60  (-3.52 – 4.56) | 1.07 ± 0.82  (-0.31 – 2.42) | 0.80 ± 1.01  (-1.88 – 2.85) | F=0.286, p=0.75 |
| Normal-appearing white matter | 0.25 ± 0.91  (-2.39 – 2.01) | 0.12 ± 0.86  (-2.39 – 1.97) | 0.36 ± 1.10  (-1.43 – 2.01) | -0.29 ± 0.83  (-1.21 – 1.81) | F=0.400, p=0.67 |
| White matter hyperintensity | 0.792 ± 1.05  (-2.39 – 3.14) | 0.79 ± 1.32  (-2.39 – 3.14) | 0.60 ± 0.97  (-1.19 – 2.41) | 0.92 ± 0.84  (-1.57 – 2.63) | F=0.486, p=0.62 |
| **Plasma volume (*v_P_*_,_ 10^-2^)** | | | | | |
| Subcortical grey matter | 1.27 ± 0.28  (0.62 – 1.79) | 1.37 ± 0.20  (1.07 – 1.74) | 1.31 ± 0.30  (0.81 – 1.77) | 1.17 ± 0.29  (0.62 – 1.79) | F=3.73, p=0.029 |
| Normal-appearing white matter | 0.55 ± 0.18  (0.12 – 0.96) | 0.63 ± 0.17  (0.35 – 0.96) | 0.63 ± 0.17  (0.39 – 0.92) | 0.44 ± 0.15  (0.12 – 0.73) | F=12.1, p<0.0001 |
| White matter hyperintensity | 0.67 ± 0.28  (0.21 – 1.76) | 0.82 ± 0.25  (0.24 – 1.51) | 0.69 ± 0.33  (0.22 – 1.76) | 0.54 ± 0.21  (0.21 – 1.16) | F=7.30, p=0.0014 |
| **Phase contrast MRI** | | | | | |
| Arterial pulsatility index | 1.25 ± 0.35  (0.56 – 2.90) | 1.37 ± 0.48  (0.69 – 2.90) | 1.06 ± 0.21  (0.56 – 1.41) | 1.27 ± 0.25  (0.89 – 2.04) | F=4.91, p=0.010 |
| Internal carotid pulsatility index | 1.27 ± 0.42  (0.57 – 3.48) | 1.43 ± 0.60  (0.66 – 3.48) | 1.08 ± 0.27  (0.57 – 1.87) | 1.27 ± 0.28  (0.85 – 2.00) | F=3.87, p=0.025 |
| Vertebral artery pulsatility index | 1.34 ± 0.35  (0.54 – 2.55) | 1.38 ± 0.39  (0.80 – 2.55) | 1.11 ± 0.23  (0.54 – 1.54) | 1.45 ± 0.32  (0.92 – 2.10) | F=7.18, p=0.0014 |
| Internal jugular vein pulsatility index | 1.09 ± 0.49  (0.40 – 2.57) | 1.05 ± 0.41  (0.40 – 1.92) | 1.03 ± 0.55  (0.41 – 2.57) | 1.17 ± 0.51  (0.49 – 2.08) | F=0.59, p=0.56 |
| Transverse sinus pulsatility index | 0.50 ± 0.18  (0.13 – 1.10) | 0.58 ± 0.20  (0.29 – 1.10) | 0.50 ± 0.17  (0.27 – 0.78) | 0.42 ± 0.14  (0.13 – 0.80) | F=6.39, p=0.0028 |
| Superior sagittal sinus pulsatility index | 0.47 ± 0.18  (0.10 – 0.95) | 0.56 ± 0.18  (0.21 – 0.95) | 0.46 ± 0.18  (0.22 – 0.71) | 0.41 ± 0.15  (0.10 – 0.76) | F=5.01, p=0.0091 |
| Straight sinus pulsatility index | 0.46 ± 0.17  (0.13 – 0.81) | 0.55 ± 0.17  (0.16 – 0.81) | 0.45 ± 0.16  (0.19 – 0.74) | 0.40 ± 0.15  (0.13 – 0.78) | F=6.24, p=0.0031 |
| Cerebrospinal fluid stroke volume at foramen magnum (ml) | 0.57 ± 0.24  (0.10 – 1.64) | 0.66 ± 0.29  (0.24 – 1.64) | 0.50 ± 0.23  (0.10 – 0.96) | 0.55 ± 0.20  (0.11 – 0.85) | F=2.67, p=0.076 |
| **Cerebrovascular reactivity magnitude (%/mmHg)** | | | | |  |
| Subcortical grey matter | 0.127 ± 0.072  (-0.182 – 0.237) | 0.114 ± 0.086  (-0.182 – 0.237) | 0.160 ± 0.039  (0.085 – 0.212) | 0.121 ± 0.071  (-0.101 – 0.220) | χ^2^=5.82, p=0.055 |
| Normal-appearing white matter | 0.035 ± 0.036  (-0.128 – 0.093) | 0.029 ± 0.045  (-0.128 – 0.086) | 0.043 ± 0.013  (0.030 – 0.075) | 0.035 ± 0.036  (-0.058 – 0.093) | χ^2^=0.612, p=0.74 |
| White matter hyperintensity | 0.022 ± 0.071  (-0.284 – 0.167) | 0.019 ± 0.093  (-0.284 – 0.109) | 0.027 ± 0.066  (-0.076 – 0.167) | 0.021 ± 0.056  (-0.138 – 0.114) | χ^2^=0.969, p=0.62 |

**Supplemental Table 6**: Additional summary statistics for the phase contrast data by disease subtype.

|  | **All patients** | **Sporadic SVD** | **CADASIL** | | **CADASIL vs sporadic SVD** |
| --- | --- | --- | --- | --- | --- |
| **Phase contrast MRI** | | | | | |
| Internal carotid pulsatility index | 1.27 ± 0.42  (0.57 – 3.48) | 1.27 ± 0.50  (0.57 – 3.48) | | 1.27 ± 0.28  (0.85 – 2.00) | t=0.03, p=0.98 |
| Vertebral artery pulsatility index | 1.34 ± 0.35  (0.54 – 2.55) | 1.25 ± 0.35  (0.54 – 2.55) | | 1.45 ± 0.32  (0.92 – 2.10) | t=-2.52, p=0.014 |
| Internal jugular vein pulsatility index | 1.09 ± 0.49  (0.40 – 2.57) | 1.04 ± 0.48  (0.40 – 2.57) | | 1.17 ± 0.51  (0.49 – 2.08) | t=-1.08, p=0.29 |
| Transverse sinus pulsatility index | 0.50 ± 0.18  (0.13 – 1.10) | 0.55 ± 0.19  (0.27 – 1.10) | | 0.42 ± 0.14  (0.13 – 0.80) | t=3.29, p=0.0015 |
| Straight sinus pulsatility index | 0.46 ± 0.17  (0.13 – 0.81) | 0.50 ± 0.17  (0.16 – 0.81) | | 0.40 ± 0.15  (0.13 – 0.78) | t=2.83, p=0.0061 |

**Supplement Table 7**: Linear mixed models with tissue based vascular dysfunction metrics as outcome metrics adjusting for the remaining vascular dysfunctions and key risk factors with arterial pulsatility substituting venous pulsatility. 95% CI=95% confidence interval, WMH=white matter hyperintensity, ICV=intracranial volume.

|  | **Outcome** | **Predictor** | **Estimate** | **95% CI** | **p value** |
| --- | --- | --- | --- | --- | --- |
| a) | *PS* | Intercept | 0.52 | -1.13 – 2.17 | 0.53 |
|  |  | Age | -0.0058 | -0.0268 – 0.0153 | 0.59 |
|  |  | Smoker | -0.087 | -0.567 – 0.393 | 0.72 |
|  |  | log_10_(WMH volume)/ICV | 0.192 | -0.276 – 0.660 | 0.41 |
|  |  | Arterial pulsatility index | -0.084 | -0.780 – 0.612 | 0.81 |
|  |  | Systolic BP variability | 7.9 | -4.4 – 20.2 | 0.20 |
|  |  | *CVR* magnitude | -0.43 | -4.26 – 3.40 | 0.82 |
|  |  | Tissue | 0.48 | -0.17 – 1.12 | 0.14 |
|  |  | log10normwmh*Tissue | -0.043 | -0.367 – 0.281 | 0.79 |
| b) | *v_P_* | Intercept | 0.166 | -0.108 – 0.441 | 0.23 |
|  |  | Age | 0.00170 | -0.00173 – 0.00512 | 0.33 |
|  |  | Smoker | -0.0172 | -0.0949 – 0.0605 | 0.66 |
|  |  | log_10_(WMH volume)/ICV | -0.110 | -0.190 – -0.029 | 0.0086 |
|  |  | Arterial pulsatility index | -0.094 | -0.207 – 0.018 | 0.099 |
|  |  | Systolic BP variability | 2.22 | 0.23 – 4.21 | 0.030 |
|  |  | *CVR* magnitude | 0.81 | 0.07 – 1.55 | 0.033 |
|  |  | Tissue | -0.046 | -0.199 – 0.107 | 0.549 |
|  |  | log10normwmh*Tissue | -0.089 | -0.166 – -0.012 | 0.024 |
| c) | *CVR* | Intercept | 0.087 | 0.011 – 0.163 | 0.026 |
|  |  | age | -0.00074 | -0.00173 – 0.00024 | 0.13 |
|  |  | smoker | -0.0037 | -0.0264 – 0.0188 | 0.74 |
|  |  | log_10_(WMH volume)/ICV | -0.0130 | -0.0351 – 0.0092 | 0.25 |
|  |  | Arterial pulsatility index | -0.0007 | -0.0335 – 0.0321 | 0.97 |
|  |  | Systolic BP variability | -0.383 | -0.962 – 0.196 | 0.19 |
|  |  | *PS* (x10000) | -0.00061 | -0.00970 – 0.00848 | 0.89 |
|  |  | Tissue | -0.0487 | -0.0801 – -0.0173 | 0.0029 |
|  |  | log10normwmh*Tissue | -0.0210 | -0.0368 – -0.0052 | 0.010 |

**Supplemental Table 8**: SVDs@Target collaborators

| **Study Sites** | **Function** | **Facility name, address** |
| --- | --- | --- |
| **University of Edinburgh, United Kingdom** | | |
| Joanna Wardlaw | Steering Committee Member;  Principal Investigator; Others (supervision of blinded image analysis and processing) | Centre for Clinical Brain Sciences,  49 Little France Crescent,  Edinburgh EH16 4SB  United Kingdom |
| Fergus Doubal | Sub-Investigator |  |
| Gordon Blair | Sub-Investigator |  |
| Carmen Arteaga Reyes | Sub-Investigator; Others (performed blinded structural MRI ratings) |  |
| Daniela Jamie Garcia | Sub-Investigator; Others (performed blinded structural MRI ratings) |  |
| Michael Stringer | Others (physicist; image acquisition and analysis including CVR) |  |
| Francesca Chappell | Others (statistician, data management and analysis) |  |
| Agniete Kampaite | Others (checked accuracy of image tissue masks) |  |
| Michael Thrippleton | Others (physicist; image acquisition and analysis including T_1_ and DCE) |  |
| Iona Hamilton | Others (study coordination) |  |
| Una Clancy | Others (study coordination) |  |
| Rosalind Brown | Others (study coordination) |  |
| **LMU Munich, Germany** | | |
| Martin Dichgans | Steering Committee member; Principal Investigator | Institute for Stroke and Dementia Research,  Feodor-Lynen-Str. 17,  81377 Munich  Germany |
| Anna Kopczak | Sub-Investigator |  |
| Marco Duering | Sub-Investigator |  |
| Daniel Janowitz | Sub-Investigator |  |
| Maria Kaffe | Sub-Investigator |  |
| Benno Gesierirch | Others (performed MRI scans) |  |
| Michael Ingrisch | Others (MRI scan setup; analysis support) |  |
| Sandra Hein | Research Nurse |  |
| Mathias Hübner | Others (performed MRI scans) |  |
| Karin Waegemann | Others (study coordination) |  |
| Anna Dewenter | Others (performed dMRI analysis) |  |
| Rainer Malik | Others (data analysis) |  |
| **University of Maastricht, The Netherlands** | | |
| Robert van Oostenbrugge | Steering Committee Member;  Principal Investigator | Department of Neurology,  University Medical Centre |
| Julie Staals | Sub-Investigator | Maastricht, |
| Danielle Kerkhofs | Sub-Investigator | P. Bebyelaan 25, |
| Maud van Dinther | Sub-Investigator | 6229 HX Maastricht |
| Walter Backes | Others (physicist, MRI setup) | The Netherlands |
| Eefje Cleophas | Research analyst |  |
| **University of Oxford, United Kingdom** | | |
| Peter Rothwell | Principal Investigator | Nuffield Department of Clinical Neurosciences,  John Radcliffe Hospital,  Oxford OX3 9DU  United Kingdom |
| Alastair Webb | Steering Committee Member; Principal Investigator |  |
| Karolina Wartolowska | Sub-Investigator |  |
| Louise Silver | Research Nurse |  |
| Josie Brooks | Research Nurse |  |
| **University of Utrecht, The Netherlands** | | |
| Geert Jan Biessels | Steering Committee Member;  Principal Investigator | Department of Neurology,  UMC Utrecht Brain Centre,  Universiteitsweg 100,  3584 CG Utrecht  The Netherlands |
| Hilde van den Brink | Sub-Investigator |  |
| Laurien Onkenhout | Sub-Investigator |  |
| Jeroen Siero | Others (performed CVR setup) |  |
| Jeroen Hendrikse | Others (performed MRI scans) |  |
| Tine Arts | Others (performed MRI scans) |  |
| Jaco JMZ Zwanenburg | Others (scanning and analysis) |  |
| Stanley DT Pham | Others (analysis) |  |
| Manja Litjens | Others (study coordination) |  |

**References**

1. Weinreb J, Wilcox PA, Hayden J, Lewis R, Froelich J. Acr mri accreditation: Yesterday, today, and tomorrow. *J Am Coll Radiol*. 2005;2:494-503

2. Radiology ACo. Site scanning instructions for use of the mr phantom for the acr mri accreditation program. *Reston, VA: American College of Radiology*. 2000

3. Thrippleton MJ, Shi Y, Blair G, Hamilton I, Waiter G, Schwarzbauer C, et al. Cerebrovascular reactivity measurement in cerebral small vessel disease: Rationale and reproducibility of a protocol for mri acquisition and image processing. *Int J Stroke*. 2018;13:195-206

4. Shi Y, Thrippleton MJ, Blair GW, Dickie DA, Marshall I, Hamilton I, et al. Small vessel disease is associated with altered cerebrovascular pulsatility but not resting cerebral blood flow. *J Cereb Blood Flow Metab*. 2020;40:85-99

5. Blair GW, Stringer MS, Thrippleton MJ, Chappell FM, Shuler K, Hamilton I, et al. Imaging neurovascular, endothelial and structural integrity in preparation to treat small vessel diseases. The investigate-svds study protocol. Part of the svds@target project. *Cerebral Circulation - Cognition and Behavior*. 2021;2:100020

6. Manning C, Stringer M, Dickie B, Clancy U, Valdés Hernandez MC, Wiseman SJ, et al. Sources of systematic error in dce-mri estimation of low-level blood-brain barrier leakage. *Magnetic Resonance in Medicine*. 2021;86:1888-1903

7. Jenkinson M, Beckmann CF, Behrens TE, Woolrich MW, Smith SM. Fsl. *Neuroimage*. 2012;62:782-790

8. Clayden JD, Maniega SM, Storkey AJ, King MD, Bastin ME, Clark CA. Tractor: Magnetic resonance imaging and tractography with r. *J Stat Softw*. 2011;44:1-18

9. Ritchie SJ, Bastin ME, Tucker-Drob EM, Maniega SM, Engelhardt LE, Cox SR, et al. Coupled changes in brain white matter microstructure and fluid intelligence in later life. *J Neurosci*. 2015;35:8672-8682

10. Munoz Maniega S, Chappell FM, Valdes Hernandez MC, Armitage PA, Makin SD, Heye AK, et al. Integrity of normal-appearing white matter: Influence of age, visible lesion burden and hypertension in patients with small-vessel disease. *J Cereb Blood Flow Metab*. 2017;37:644-656

11. Li X, Morgan PS, Ashburner J, Smith J, Rorden C. The first step for neuroimaging data analysis: Dicom to nifti conversion. *J Neurosci Methods*. 2016;264:47-56

12. Andersson JLR, Skare S, Ashburner J. How to correct susceptibility distortions in spin-echo echo-planar images: Application to diffusion tensor imaging. *Neuroimage*. 2003;20:870-888

13. Andersson JLR, Sotiropoulos SN. An integrated approach to correction for off-resonance effects and subject movement in diffusion mr imaging. *Neuroimage*. 2016;125:1063-1078

14. Smith SM. Fast robust automated brain extraction. *Hum Brain Mapp*. 2002;17:143-155

15. Salvador R, Pena A, Menon DK, Carpenter TA, Pickard JD, Bullmore ET. Formal characterization and extension of the linearized diffusion tensor model. *Human Brain Mapping*. 2005;24:144-155

16. Greve DN, Fischl B. Accurate and robust brain image alignment using boundary-based registration. *Neuroimage*. 2009;48:63-72

17. Thrippleton MJ, Blair GW, Valdes-Hernandez MC, Glatz A, Semple SIK, Doubal F, et al. Mri relaxometry for quantitative analysis of uspio uptake in cerebral small vessel disease. *Int J Mol Sci*. 2019;20

18. Friston KJ, Williams S, Howard R, Frackowiak RS, Turner R. Movement-related effects in fmri time-series. *Magn Reson Med*. 1996;35:346-355

19. Thrippleton MJ, Backes WH, Sourbron S, Ingrisch M, van Osch MJP, Dichgans M, et al. Quantifying blood-brain barrier leakage in small vessel disease: Review and consensus recommendations. *Alzheimers Dement*. 2019;15:840-858

20. Jenkinson M, Bannister P, Brady M, Smith S. Improved optimization for the robust and accurate linear registration and motion correction of brain images. *Neuroimage*. 2002;17:825-841

21. Heye AK, Thrippleton MJ, Armitage PA, Valdes Hernandez MDC, Makin SD, Glatz A, et al. Tracer kinetic modelling for dce-mri quantification of subtle blood-brain barrier permeability. *Neuroimage*. 2016;125:446-455

22. McCarthy P. Fsleyes. 2021

23. Walker PG, Cranney GB, Scheidegger MB, Waseleski G, Pohost GM, Yoganathan AP. Semiautomated method for noise-reduction and background phase error correction in mr phase-velocity data. *Jmri-J Magn Reson Im*. 1993;3:521-530

24. Bateman GA. Pulse-wave encephalopathy: A comparative study of the hydrodynamics of leukoaraiosis and normal-pressure hydrocephalus. *Neuroradiology*. 2002;44:740-748

25. Sleight E, Stringer MS, Marshall I, Wardlaw JM, Thrippleton MJ. Cerebrovascular reactivity measurement using magnetic resonance imaging: A systematic review. *Front Physiol*. 2021;12:643468
